# Opportunities and Challenges of Switchable Materials for Pharmaceutical Use

**DOI:** 10.3390/pharmaceutics14112331

**Published:** 2022-10-28

**Authors:** Deniz Ceylan Tuncaboylu, Christian Wischke

**Affiliations:** Institute of Active Polymers, Helmholtz-Zentrum Hereon, Kantstr. 55, 14513 Teltow, Germany

**Keywords:** switchable polymers, smart materials, stimuli-sensitive polymers, controlled drug-release systems

## Abstract

Switchable polymeric materials, which can respond to triggering signals through changes in their properties, have become a major research focus for parenteral controlled delivery systems. They may enable externally induced drug release or delivery that is adaptive to in vivo stimuli. Despite the promise of new functionalities using switchable materials, several of these concepts may need to face challenges associated with clinical use. Accordingly, this review provides an overview of various types of switchable polymers responsive to different types of stimuli and addresses opportunities and challenges that may arise from their application in biomedicine.

## 1. Introduction

Drug delivery aims to achieve a therapeutic effect by administering a pharmaceutical compound that becomes effective at the desired site of the body, while matching the necessary concentrations and duration of action. Particularly for parenteral applications, bio-materials are needed, which are capable of tailoring the release of the drug with controlled release rates. After the establishment of polyester-based carrier systems, which have entered clinics [1], the major research direction of switchable materials has developed; these materials have also been called “smart polymers”, “stimuli-responsive polymers”, or “environmentally sensitive polymers”, with each term having a slightly different meaning [2].

Switchable materials should offer a means to modulate biological/pharmacological effects based on an intended and typically fast alteration of the materials’ properties. However, to the present day, the polymers under consideration are unable to perform intelligent information processing and decision making, which is why the term “smart materials” has occasionally been criticized [3]. Instead, the response can be governed by conformational rearrangements, phase transitions, the cleavage of chemical bonds, catalytic reactions, or a combination of them, which manifest as alterations of superordinated physical properties such as changes in surface characteristics, solubility, volume, or shape, or the formation/disturbance of molecular assemblies. In some cases, smart polymers can experience physical or chemical changes that are reversible, thus allowing them to respond several times to small variations in environmental conditions. Typical arrangements of polymers are, e.g., (i) linear free chains in solution, where the polymer undergoes a reversible collapse after an external stimulus is applied; (ii) covalently cross-linked polymer networks, where swelling or shrinking of the gels occurs by aid of the trigger; and (iii) chains adsorbed or grafted to a surface, where the chains expand or coil on the surface, thus changing the density and interaction capabilities [4,5]. In an idealized situation, a carrier or device based on such materials would be able to recover to its initial state when the exposure to the stimulus ends or a second stimulus is applied [6].

In pharmaceutics, switchable materials may need to fulfill further requirements beyond the switching capability in order to serve as matrix materials for drug carriers. The most important are:

- The materials and their stimuli should be compatible with physiological requirements. However, this putatively trivial fact is not considered in numerous publications, suggesting the biomedical use of materials that are responsive only to, e.g., intense irradiation, non-physiological solvents, or high temperatures, or are based on components that are expected to show short-term or long-term toxicity.
- The drug should be effectively incorporated in relevant quantities, with material switching allowing the adjustment of drug-release rates, while premature diffusion-controlled release is suppressed. Typically, drug release should be enhanced upon stimulation. The inhibition of ongoing release from an implanted long-term drug-dosage system might also be of interest, e.g., in case of critical side effects of the medication or to adapt release rates to the progress of healing processes or physiological cycles.
- The in vivo fate of the carrier materials should be considered. While non-degradable large-sized devices may be surgically removed, this is typically not comfortable for patients. In the case of injectables based on particulate carriers, surgical removal is practically impossible, thus setting specific requirements for degradability, suitability for excretion, and/or cellular clearance. Theoretical degradability of some or all bonds in a polymeric construct does not necessarily mean that the material can or will be quantitatively removed in the expected time frame. For instance, a solubility drop of hydrophobic segments after cleavage from amphiphilic copolymers, or crystallization upon increasing the chain mobility of oligomeric degradation products, may create long-lasting residues.

Most explored drug carriers based on switchable materials follow one of the following two principles: (i) an all-at-a-time approach, such as via container systems, which burst or degrade, and thus, do not allow the drug release to be stopped once initiated, and (ii) systems that accelerate drug release only at the time of exposure to environmental changes, which requires not only reversibility of the polymeric switches but also integrity of the carrier itself.

**Figure 1:**
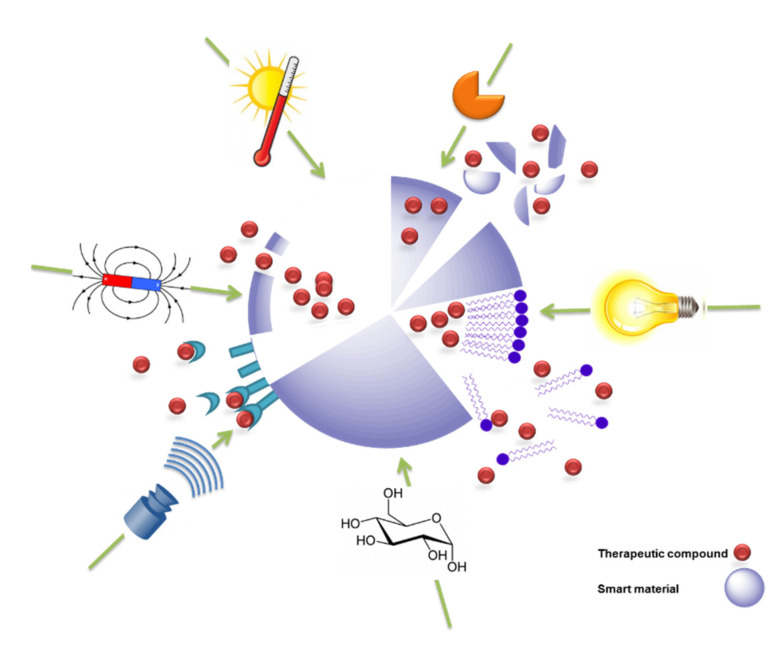
Schematic illustration of switchable drug-release systems. The release of therapeutics is triggered from a model drug carrier by exposure to the respective stimulus/stimuli, including temperature, enzymes, light, ultrasound, magnetic field, or small molecules such as glucose.

The stimuli that can be exploited to trigger the release of a therapeutic or theranostic agent from a drug-delivery system (DDS) are diverse (Figure 1), but can be classified into two major types: externally applied versus in vivo stimuli. Externally controlled systems rely on triggers applied from outside the biological system. The stimuli should be distinguished, either in their nature (e.g., the application of alternating magnetic fields) or their extent (e.g., the level of temperature), from signals within the body during (patho-) physiological processes. In case of systems with an in vivo regulation without external interference, the triggers need to originate from the cellular or interstitial environment of a drug carrier. If the carrier systems contain reversible switches, this, ideally, may lead to release rates being susceptible to feedback mechanisms that occur naturally in target cells or tissues. Accordingly, such release systems may be autonomous and could exhibit an adapted drug release.

Having in mind this vision of future drug-carrier systems, this review aims to critically discuss existing concepts of switchable materials and their potential contribution to future drug-release systems. In particular, challenges may need to be considered that arise from medical application, such as the types of stimuli that can be realistically applied. Accordingly, the article will be structured based on the different types of stimuli and will also explore the physical basis behind these effects. Some polymers and functions, which have not yet been studied within the settings of drug delivery but show potential for this application, will also be briefly discussed.

## 2. Responsiveness to External Stimuli

### 2.1. Temperature-Responsive Materials

Temperature-responsive polymers are the most extensively studied class of switchable polymeric systems. They served as models to demonstrate how stimuli-responsive properties can be modulated and utilized for drug-delivery technology [7,8,9,10]. Heat is considered here as an external stimulus, despite the fact that small temperature differences can also occur in the body during pathophysiological conditions such as fever or local infections. Additionally, tumor microenvironments are often 1−2 °C warmer than normal tissues [11]. However, facilitating such small temperature differences for targeted drug delivery can be considered a major challenge. Therefore, in many cases, externally induced and locally administered hyperthermia in a target area is explored more extensively as a trigger than small pathophysiological temperature differences. Thermosensitive materials are based on a number of different switching mechanisms, which provide characteristic features of responsive materials and carrier systems.

*Swollen polymers with miscibility gaps.* Very prominent temperature-responsive materials are polymers that exhibit either a lower critical solution temperature (LCST) and/or an upper critical solution temperature (UCST). These polymers are completely miscible with a given solvent at conditions below (LCST) or above (UCST) a critical temperature, but become (partially) insoluble, e.g., above the LCST [12]. It is important to note that those materials typically do not undergo chemical alterations causing changes in hydrophobicity/hydrophilicity, but instead, experience a temperature-dependent shift in the relative ratio of hydrophobic/hydrophilic interactions within the polymer and with the surrounding environment/solvent [13]. Thermodynamically, below the LCST, the enthalpy term is responsible for polymer solubility, which is related to the hydrogen bonding capabilities of the constituent monomer units. Above the LCST, hydrophobic interactions dominate, leading to polymer precipitation. It is noteworthy that the LCST is an entropically driven effect, while the UCST is driven by enthalpy [14]. A change in temperature, for a given polymer concentration, can shift the system towards the miscibility gap, resulting in gelation or shrinkage that also becomes visible on a macroscopic level [15].

In pharmaceutical sciences, polymers with LCST are more relevant than UCST systems. The therapeutic agents, such as drugs, cells or proteins, can be mixed with the polymer solution at a low temperature, forming a gel when warmed up to body temperature after injection [16]. From this depot, drug release may typically be diffusion-controlled. However, in contrast to other in situ forming depot systems based on organic polymer solutions that precipitate upon solvent extraction [17], the reversibility of LCST transitions may allow liquefication of the matrix by applying local hypothermia to the tissue (e.g., <35 °C, [18]), thus possibly enhancing drug-diffusion rates.

**Figure 2:**
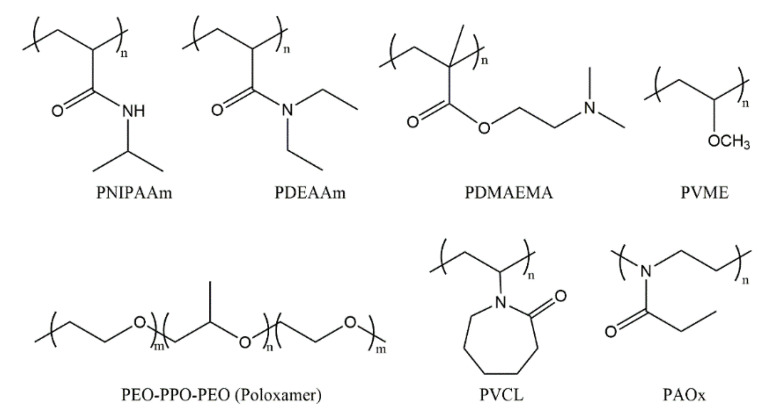
Schematic structure of selected thermoresponsive polymers that show an LCST. PNIPAAm: poly(*N*-isopropylacrylamide), PDEAAm: poly(*N*,*N*-diethylacrylamide), PDMAEMA: poly[2-(dimethylamino)ethyl methacrylate], PVME: poly(vinyl methyl ether), PEO-PPO-PEO: poly(ethylene oxide)-*b*-poly(propylene oxide)-*b*-poly(ethylene oxide) with the international nonproprietary name Poloxamer, PVCL: poly(*N*-vinylcaprolactam), PAOx: poly(2-alkyl-2-oxazoline).

Two classes of polymers with an LCST have been widely investigated in the field of pharmaceutics: poly(*N*-isopropylacrylamide) (PNIPAAm)-based polymers [19] and amphiphilic block copolymers based on poly(ethylene oxide) (PEO) and poly(propylene oxide) (PPO), or poly(ethylene glycol) (PEG) and poly(lactic acid) (PLA) segments. In addition to them, other polymers with thermoresponsive properties include poly(*N*,*N*-diethylacrylamide) (PDEAAm) with an LCST in the range of 25 to 32 °C [20], poly(*N*-vinylcaprolactam) (PVCL) [21,22,23] with an LCST between 25 and 35 °C, poly(vinyl methyl ether) (PVME) [24,25] with an LCST of about 36 °C, poly[2-(dimethylamino)ethyl methacrylate] (PDMAEMA) with an LCST of around 40 °C [26], and poly(2-alkyl-2-oxazoline)s (PAOxs) [27] (Figure 2).

Despite not being freely adjustable for a given polymer system, the LCST depends on the molecular weight and architecture of the respective polymer, including the size of the side groups. Thus, the LCST can be “tuned” by variation of the hydrophilic or hydrophobic moieties [19,28,29,30,31] or by copolymerization with other monomers to obtain an LCST close to (typically a few Kelvin below) physiological temperature for applications in drug delivery. For example, the nature of the alkyl substituent in the 2-position of the 2-oxazoline monomer determines the relative hydrophobicity, and thus, the LCST of the resulting polymer. PAOxs with methyl side chains are very hydrophilic and do not show a phase transition in water, but extending the hydrophobic side-chain length to ethyl or propyl leads to an LCST of about 25 °C [28]. More control over LCST can be achieved with variation in the propyl side chain; poly(2-oxazolines) with isopropyl and cyclopropyl side chains have LCST values of ~35 °C and ~30 °C, respectively [29]. Furthermore, various copolymers and molecular architectures of PAOx-containing materials have been studied [30], and their applications in drug delivery beyond temperature sensitivity [31], e.g., for stealth properties [32], have been investigated extensively.

In the following, a number of LCST-based carrier systems will be described. PPO/PEO-based triblock copolymers (poloxamers) are listed as additives in the *Pharmacopoeia*, which distinguishes poloxamers from most other experimental materials, and are accepted in clinical use [10]. At high concentrations, i.e., typically > 20 wt.%, depending on the respective block length and total molecular weight, dehydration and enhanced hydrophobic interactions of PPO blocks can lead to the formation of micellar associations that cause gelation [33]. Different models have been proposed to describe this phenomenon of triblock copolymers, such as percolated micellar networks [34] or the jammed micelle model [35]. While the macroscopic sol–gel transition of poloxamer solutions can be recalled numerous times, the system does not return to its initial state on the molecular level because of high solution viscosity and slow relaxation of intermicellar entanglements [33]. The in situ gelation of poloxamers is a common strategy in mucosal drug delivery, e.g., for increased retention of liquid formulations in the nasal cavity or in the eye [36,37]. 

A major hurdle of poloxamer gels is their typically poor mechanical stability upon immersion into excess water [35]. The mechanical properties of poloxamer hydrogels were improved when mixed micelles were prepared from thermosensitive poloxamers together with diblock copolymers containing photocrosslinkable coumarin molecules in their repeating units [38]. Furthermore, enhanced mechanical stability was observed for poloxamer-based injectable hydrogels prepared in the presence of hyaluronic acid (HA) and cyclodextrin (CD) molecules, where HA chains and CD units increased secondary interactions, while more therapeutic molecules could be loaded through CD molecules [39]. The stability can also be enhanced by hydrophobic segments that exhibit stronger physical interactions, e.g., block copolymers of PEO with poly(*L*-lactic acid) (PLLA) or poly[(*rac*-lactic acid)-*co*-glycolic acid) (PLGA), which additionally introduce the capability for hydrolytic degradation into the polymer [40,41]. PLGA/PEO-based di-/triblock copolymers, again, depending on molecular weight and the PEO-PLGA ratio [42], can from transparent gels with maintained structural integrity and mechanical strength, which present a sol–gel transition at ~30 °C [43]. Such block polymers have been used for the release of both hydrophilic and hydrophobic drugs, as well as for protein encapsulation [44,45,46,47]. In addition to PEO, other hydrophilic polymers with suitable hydrophobic substituents can show thermoresponsiveness, e.g., linear or branched polyglycerols, where the free hydroxyl groups can be grafted with short alkyl groups [48].

**Figure 3:**
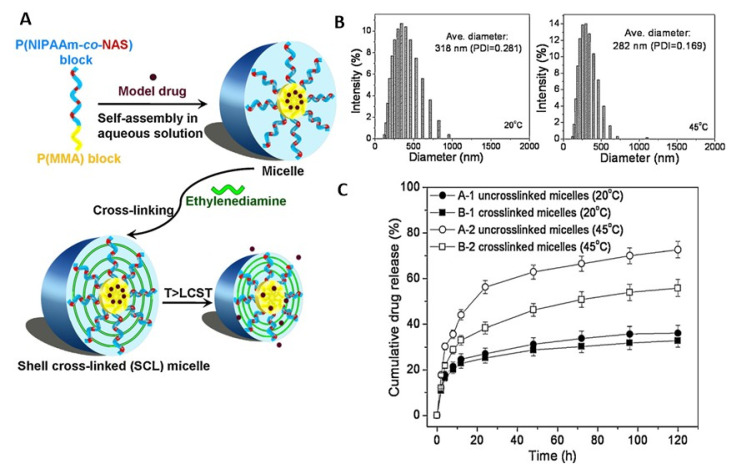
(**A**) Schematic illustration of the structure and switching capability of shell-crosslinked micelles from block copolymers of polymethyl methacrylate and poly(*N*-isopropylacrylamide-*co*-*N*-acryloxysuccinimide). (**B**,**C**) Temperature-controlled alteration of drug release from the micelles in aqueous solution was accompanied with shift in the particle size distribution upon heating. Reprinted from [55], © 2022 Elsevier B.V. All rights reserved, with permission from Elsevier.

**Figure 4:**
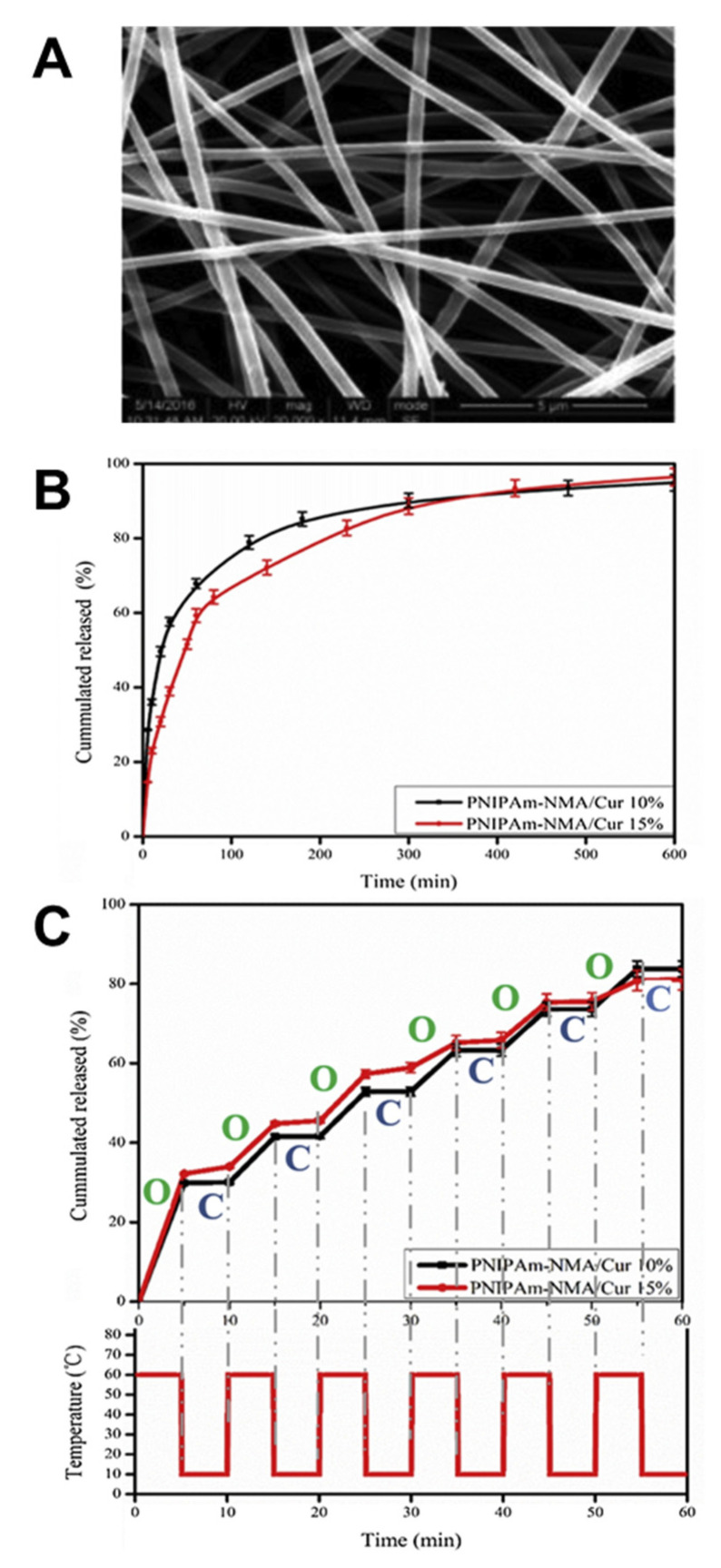
Curcumin release from crosslinked thermoresponsive poly(*N*-isopropylacrylamide-co-*N*-methylolacrylamide) fibers. (**A**) Fiber mesh prepared via electrospinning. (**B**) Release pattern at 37 °C. (**C**) Stepwise release pattern at alternating temperatures. Reprinted from [56], © 2022 Elsevier B.V. All rights reserved, with permission from Elsevier.

As another type of polymer with an LCST, PNIPAAm appears to be one of the most extensively investigated thermosensitive materials [2]. PNIPAAm typically exhibits a phase transition at 32 °C in water, which can be adjusted (e.g., up to 40 °C) by incorporating hydrophilic or hydrophobic comonomers [49,50]. In addition, obtaining polymers with varied LCST from the same monomer via changes in polymer architecture is also possible [51]. Branched PNIPAAm exhibited decreased LCST compared with linear PNIPAAm (27 °C vs. 33 °C) due to the enhanced intrachain H-bonding in the branched polymer [52]. PNIPAAm-based series of disk-shaped hydrogels with an interpenetrating network structure were loaded with indomethacin and allowed to obtain on–off drug-release profiles in vitro in response to a stepwise temperature change [53,54]. In another example, anti-inflammatory hydrophobic prednisone acetate was entrapped in the core of a shell-crosslinked micellar carrier system from block copolymers of polymethyl methacrylate and poly(*N*-isopropylacrylamide-*co*-*N*-acryloxysuccinimide) (PMMA-*b*-P(NIPAAm-*co*-NAS)). Interestingly, the release was less at 20 °C (below the LCST) when compared to 45 °C (above the LCST) [55], suggesting that structural changes in the carrier during its shrinking (above LCST), rather than potentially expected enhanced drug-diffusion rates below LCST, allowed for the highest mass transport (Figure 3). In addition to particles, other PNIPAAm-based DDS have been repeatedly investigated. PNIPAAm copolymerized with *N*-methylol acrylamide (NMA) has been crosslinked after electrospinning to nonwoven meshes (LCST of ~40 °C), which showed relatively fast diffusion-controlled release of curcumin at 37 °C (Figure 4). Switching the temperatures of the release medium between 10 and 60 °C, which is outside the range that may be realized in vivo, allowed for an on–off release pattern (Figure 4) [56].

A disadvantage of PNIPAAm-based network materials may be their non-degradability under physiological conditions, which can be overcome by degradable crosslinkers such as poly(amino acids) [57]. Furthermore, PNIPAAm can be grafted onto polymers with degradable backbones such as gelatin, which was investigated for the intracameral administration of antiglaucoma medication [58]. Another critical concern may be the occasionally observed toxicity of PNIPAAm-based materials, which may possibly be attributed to residual monomers rather than the polymer itself (as supported by other studies suggesting non-toxicity [59]) and could be avoided by conducting a suitable purification procedure. It should also be noted that strong volume changes may occur upon switching for macroscopic matrices, particularly those with a low degree of crosslinking, which could create pressure on adjacent tissue. Additionally, due to structural and kinetic effects, volume changes of macroscopic samples may be slow since precipitated top-layers (above the LCST) may form a diffusion barrier to solvent transport out of the matrix core. Both of these issues can be considered to be less relevant for, e.g., PNIPAAm-based nanogel carriers.

*Multifunctional devices with shape switching.* Shape-memory polymers (SMPs) are temperature-switchable polymers that retain a practically constant volume during switching. They are based on elastic polymer network structures, are available both as hydrophobic matrices and as hydrogels, and can comprise various molecular switches, including heat-sensitive domains [60]. The SMP technology was also transferred from the level of macroscopic implants to microsized particles [61,62,63], which can be prepared from spherical stock particles via stretching deformation to ellipsoids of predefined aspect ratios [64] and may be useful for vessel occlusion and drug delivery [65]. SMPs can be deformed to a secondary shape and fixed in this state by introducing temporary netpoints, such as crystalline domains, via cooling. These temperature-sensitive switches later melt upon heating. In this way, stress stored in the material’s temporary shape is released by entropy-elastic recoil of stretched polymer chains, which eventually drives the recovery of the material to its initial macroscopic shape. This capacity may be used for the anchoring or unfolding of implants to adopt an application-relevant shape in the body [66,67]. SMP hydrogels have also been demonstrated to show a self-healing capacity after mechanical damage, which required the melting of alkyl chain crystals as crosslinks to become flexibly movable hydrophobic units that can aggregate to connect the interfaces of polymer network fragments after mechanical damage [68].

**Figure 5:**
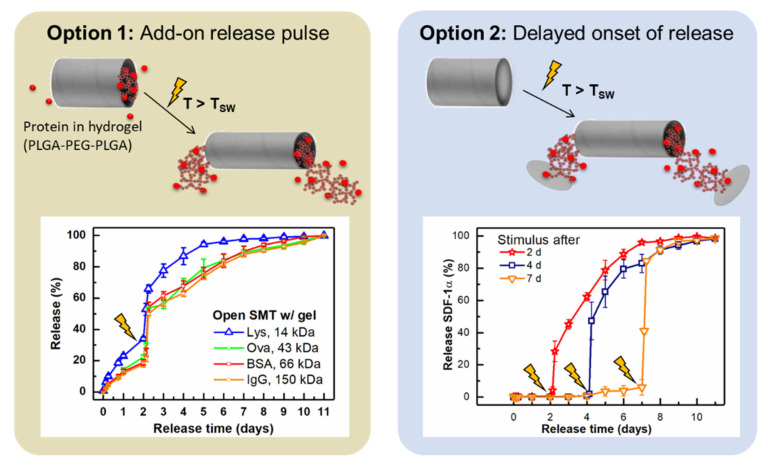
On-demand release pulse or on-demand release initiation from shape memory tubes (SMT), which switch due to heat (direct exposure or indirectly through NIR light) to smaller inner diameters and expel a hydrogel loaded with the protein of interest. Data originating from [72], © 2022 Elsevier B.V. All rights reserved, with permission from Elsevier.

Several examples of amorphous and semi-crystalline SMP networks from copoly-esters have been reported to provide a sustained drug release that is timely independent of the shape-memory function and hydrolytic degradation of these materials [69,70]. Drug release in such systems is affected, among other parameters, by the polymer composition, as seen for a series of AB copolyester networks, where increasing drug release was correlated with the amount of glycolide as the more hydrophilic repeating unit [69]. Furthermore, coating allows to control drug-diffusion rates from SMP devices, e.g., tracheal stents [71]. For on-demand release of various model proteins from SMP, container systems in the shape of tubes have been proposed. Shape switching of the tubes to smaller inner diameters was triggered by direct heat exposure or high-intensity NIR light, resulting in the ejection of the protein-loaded hydrogel from the lumen of the tube (on-demand release pulse) or the breakage of a sealing layer (on-demand release initiation) (Figure 5). Based on the modular character, the system has been proposed as a kit system to be filled with the drug of interest in the surgical theater [72].

Above the SMP’s switching temperature, enhanced diffusivity of drug molecules can be assumed, given that the polymer will be in the viscoelastic state. As also described in Section 2.4, this principle of simultaneous shape-switching and enhanced drug release was explored for copper sulfate as a model substance, where release and shape recovery were induced in a stepwise fashion with high-intensity focused ultrasound that creates heat locally [73]. Still, the concept of enhanced release from SMP via remote heating has not yet been extensively driven towards practical feasibility.

### 2.2. Light-Responsive Materials

Light of tunable intensity and wavelength can be administered with good spatial and temporal control, and thus, is an interesting external stimulus; it is less prone to rate limitations that apply in the case of heat transfer processes or the diffusion of specific molecules serving as in vivo stimuli, respectively. For medical applications, the radiation wavelength should be above ~380 nm to avoid adverse effects to the skin [74]. Visible light may be a trigger for topical treatments only as it cannot penetrate deeply into tissue because of strong scattering and absorption by water, lipids, and biomolecules (e.g., hemoglobin). However, the optical window in the near-infrared (NIR) light range (700–1100 nm) allows deeper tissue penetration. Furthermore, compared to classical one-photon processes, typically applicable to chromophores absorbing in the UV and Vis ranges, two-photon absorption of NIR light may provide a similar light energy to UV/Vis to activate photoreactions without being detrimental to tissues and cells.

**Figure 6:**
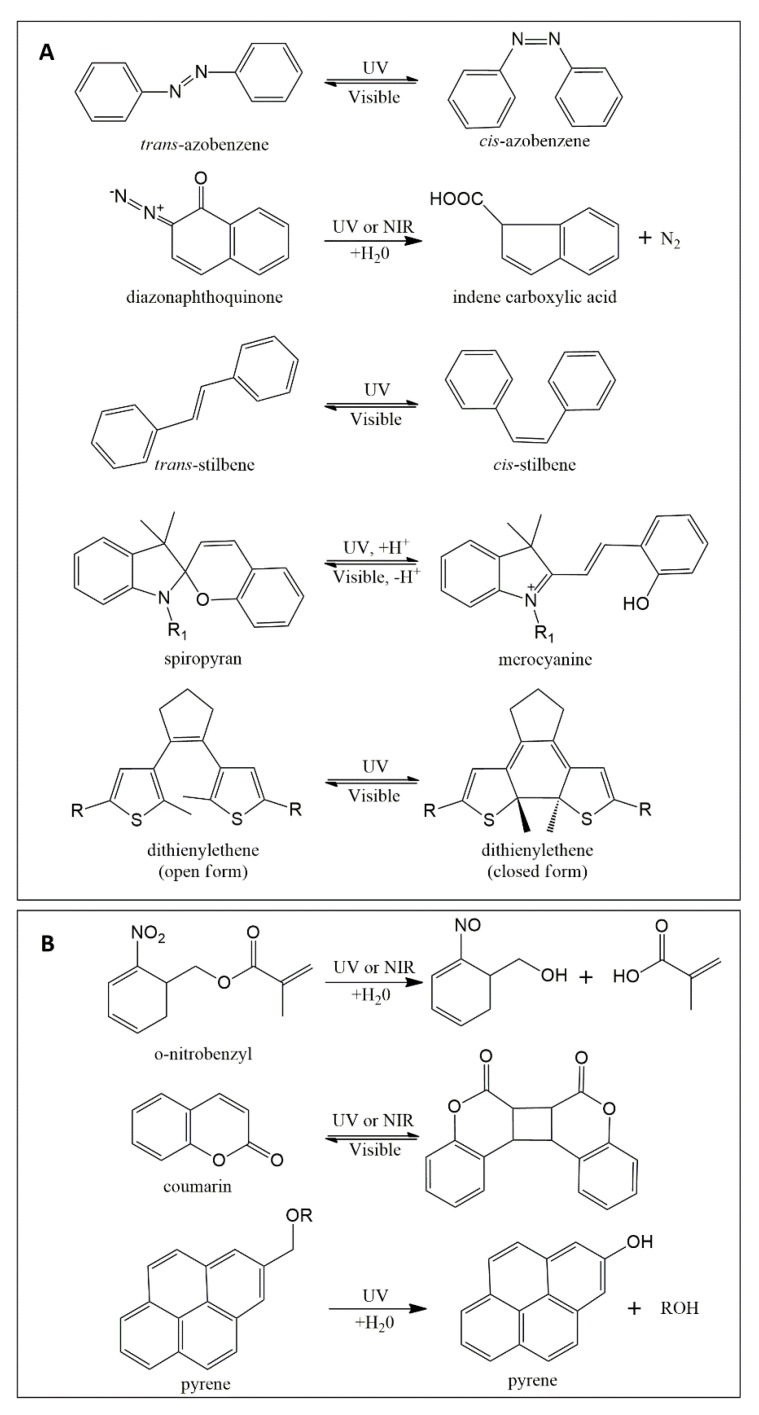
Chemical structures of selected photochromic molecules. (**A**) Moieties shifting their hydrophobicity/hydrophilicity balance and/or orientation upon irradiation. (**B**) Exemplary moieties that undergo photocleavage/photocoupling upon irradiation.

Light-responsive switchable materials can be categorized into two main groups, namely, either systems experiencing shifts in hydrophobicity/hydrophilicity or materials that undergo photocleavage reactions (for exemplary moieties, see Figure 6). Photosensitive carriers such as micelles, hydrogels, dendritic/hyperbranched polymers, polymer capsules, and supramolecular assemblies can be obtained.

*Shifts in hydrophobicity/hydrophilicity.* Materials with light-induced alteration of hydrophobicity/hydrophilicity often are amphiphilic block copolymers, in which a photochemical reaction increases the polarity of the hydrophobic block, and thus, leads to destabilization and disassembly of the carrier structure in the presence of a suitable solvent such as water. If the photoreaction is reversible (e.g., upon exposure to light of a different wavelength), the initial balance of hydrophobicity/hydrophilicity can be restored and polymers might reassemble to their initial structure (e.g., a micelle).

Different photochemical mechanisms apply for commonly employed molecular switches such as azobenzene (AZO), spiropyran (SP), dithienylethene (DTE), and diazonaphthoquinone (DNQ), and stilbene (Figure 6A), which eventually results in altered hydrophilicity. While the process for AZO and stilbene is a reversible *trans*-*cis* photoiso-merization [75], illumination of the SP molecule causes a reversible reaction from a closed ring to an open charged merocyanine [76]. DTE derivatives, widely investigated for their excellent thermal stability and rapid photo-response, need UV light to switch from open form to closed form, while the reverse process relies on visible light [77,78]. Lastly, DNQ displays a photoinduced irreversible Wolff rearrangement reaction [79] with a loss of nitrogen, yielding an indene carboxylate [80]. In some examples, the light-induced release of embedded molecules from polymers containing photoswitches was only functional after grinding, probably due to low light penetration [81]. Thus, light/drug permeable materials such as hydrogels [82], small-sized polymer matrices such as nanoparticles/-capsules [83], vehicles with light-absorbing probes located at the particle surface [84,85], or water-soluble conjugates of drugs and photocleavable targeting constructs [86] may be more feasible approaches for light-triggered release systems.

**Figure 7:**
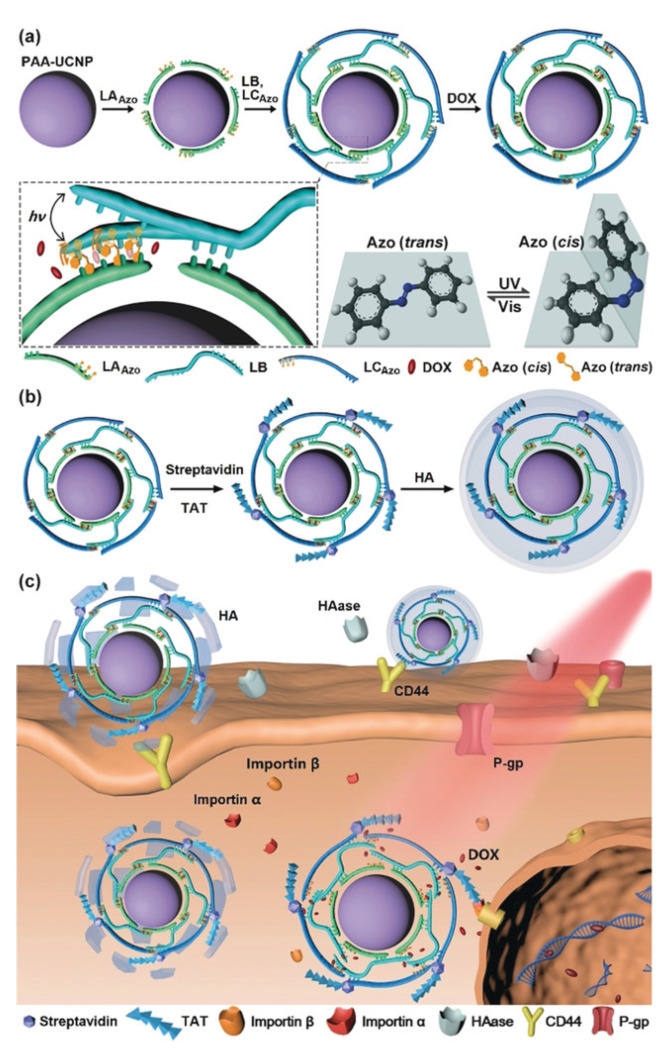
Azobenzene-based photoswitches combined with upconversion nanoparticles (UCNP; NaYF_4_:Tm,unYb functionalized with polyacrylic acid (PAA)) to release doxorubicin after dissociation of surface-bound DNA strands. (**a**) Principles of the assembly of components to form the particle cores. (**b**) Surface conjugation of TAT and HA coating. (**c**) HA-mediated cell binding and subsequent endocytosis. LA_AZO_/LC_AZO_: DNA strand with AZO moieties; LB: DNA strand; DOX: doxorubicin intercalated between the DNA strands; HA: anionic hyaluronic acid for triggered endocytosis; HAase: hyaluronidase; TAT: nuclear localization peptide. Reprinted with permission from [93], © 2022 Wiley-VCH Verlag GmbH & Co. KGaA, Weinheim.

Following the concept of polymer therapeutics, AZO-substituted poly(acrylic acid) (PAAc) has been suggested for binding of α-cyclodextrin–drug conjugates by AZO inclusion and the photoinduced drug release, e.g., of doxorubicin derivatives [87]. These conjugates would, from a legal perspective, need to be rated as new active pharmaceutical ingredient. The molecular *trans*–*cis* movement of AZO moieties located in particle shells in proximity to bioactive molecules has also been suggested to enhance compound release by acting as a molecular stirrer [88,89], while other driving principles, including thermal effects or altered non-covalent bonds, might also need to be considered. Furthermore, host/guest complexes formed between CD and *trans*-AZO, but not with *cis*-AZO, have facilitated various photocleavable supramolecular hydrogels, particles, etc. [90]. Given the light absorption of AZO in the UV range, which may not be feasible in most in vivo settings, the research focus on AZO-based switchable materials has been expanded to its use as cleavable unit, e.g., in enzyme- or hypoxia-sensitive vehicles [91,92]. Furthermore, altered ring substitution [90] and combinations with upconversion nanoparticles (UCNP) were studied for triggering AZO photoswitches at more suitable wavelengths (Figure 7) [93,94,95]. Increasing complexity (see Figure 7) and a potentially reduced overall drug-loading capacity may be some of the critical aspects that might need to be considered for such hybrid carriers.

**Figure 8:**
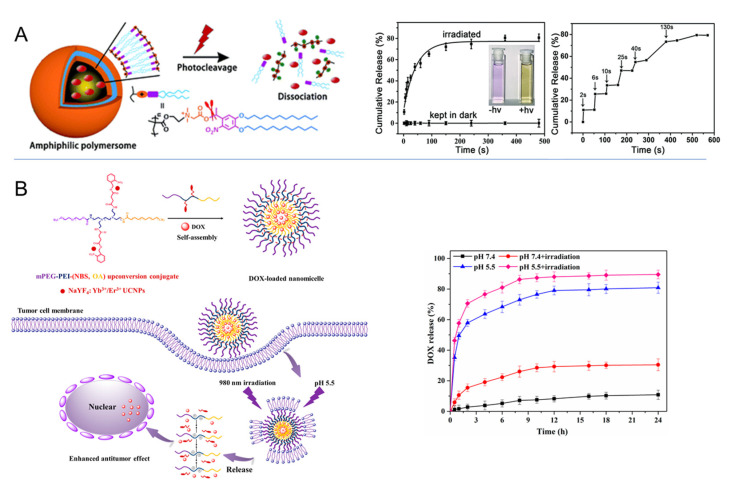
Dissociation of vesicles after photocleavage of nitrobenzyl (NB) moieties. (**A**) Cleavage of NB linker in amphiphilic shell causes dissociation of polymersome and compound release (hydrophobic dye). Used with permission of Royal Society of Chemistry, from [99]; permission conveyed through Copyright Clearance Center, Inc. (**B**) Cleavage of nitrobenzyl succinate side chain induces pH-dependent charge repulsion between polyethyleneimine (PEI) segments and micelle dissociation for compound release. Reprinted from [83]; licensed under Creative Commons Attribution (CC BY) license (http://creativecommons.org/licenses/by/4.0/), © 2022 by the authors.

*Photocleavage.* The second approach for photoswitchable materials uses photochromic moieties that can be cleaved by various photoreactions [96]. Such processes can convert a hydrophobic polymer into more hydrophilic structures, e.g., in the case of block copolymers. As a photochromic group, nitrobenzyl (NB) moieties can be positioned in the main chain or side chains of polymers or as a block junction in copolymers. The cleavage of NB is an intramolecular rearrangement process that may proceed in minutes via one-photon UV and/or two-photon NIR excitation [97]. Light irradiation leads to a nitrosobenzaldehyde byproduct, which is known to be (undesirably) reactive towards endogenous biomolecules including proteins. From the viewpoint of clinical application, the NB-containing polymers might induce toxicity in vivo. Therefore, the use of NB as cleavable linkages (at low content) between blocks/segments of (co)polymers is more suitable than NB-based unimers (containing numerous NB units). For instance, NB groups were utilized to link poly(acrylamide)−poly(ethylene glycol) (PAAm-PEG) in a hybrid gel structure loaded with UCNP, which showed a light-responsive gel−sol transition and on-demand delivery of biomacromolecules such as enzymes [98]. NB moieties were also repetitively investigated as linkers in amphiphilic molecules such as in polymersome shells (Figure 8A) [99,100], which disassociated upon NB cleavage and released their payload. The dissociation of vesicles can also be realized by pH-dependent charge repulsion after the photoinduced removal of NB-based moieties (Figure 8B) [101]. Another concept is to use physical interactions of drug molecules with NB for their retention, e.g., in microgels, where the cleavage of NB leads to increased diffusivity of the drug and the NB degradation product (see above for comment on potential toxicity) out of the gel [102].

Similar to NB, coumarins are also sensitive to one- or two-photon absorption. The coumarin family includes thousands of different derivatives, some of which have attracted extensive interest as bioactive molecules for various clinical indications [103]. Coumarin-containing polymers have been widely studied for the preparation of core or shell cross-linked micelles and nanogels, in which the dimerization reaction of coumarin can be activated by UV light [104]. Additionally, drugs such as 5-fluorouracil [105] and chlor-ambucil [106] have been coupled to coumarin moieties present as side or end groups of polymer chains, from which the drugs were released via UV-light-induced ester cleavage or reversion of a cycloaddition reaction between the drug and coumarin. The short irradiation wavelengths could become an issue in medical settings, particularly for non-fatal indications.

Pyrene derivatives coupled as a side group to a polymer can undergo a photosolvolysis reaction in the presence of water or another protonic solvent when irradiated. Block copolymers of poly(ethylene glycol)-*b*-poly(pyrene methacrylate) can form micelles, driven by the hydrophobic pyrenylmethyl ester substituents; these could be cleaved from the polymethacrylate segment upon exposure to 365 nm light, yielding a fully water-soluble polymer, and thus, dissociation of the vesicles [107]. This strategy, which is based on side-group cleavage to change polymer properties on-demand, and thereby affects carrier stability, may be a general concept that could be applicable to many polymers with suitable chromophore substituents.

*Indirect triggering of temperature sensitivity.* Beyond effects directly mediated by light exposure, as described above, light can be an indirect trigger for temperature-dependent processes via suitable absorbers (dyes, carbon particles, carbon nanotubes, and gold nanorods) that show energy dissipation. For instance, the shape-memory tubes reported above (see Figure 5) could also be activated by a pulsed NIR laser [72].

Generally, the advent of two-photon systems and NIR-responsive materials may overcome hurdles in previous works such as being limited by low wavelengths and poor in vivo penetration of UV/Vis light. However, photoreactions induced by two-photon absorption of NIR light are regarded as slower and presumably less efficient due to the low two-photon absorption cross-sections of some chromophores [108]. Another drawback is that azobenzene groups and similar groups are perceived to be toxic, which limits the application of such materials, even in topical formulations. The toxicity of the photochromic group itself and of photoreaction products thus needs to be more carefully considered when suggesting new mechanisms and release concepts for photoswitchable materials.

### 2.3. Magnetic Field-Responsive Materials

A key benefit of magnetic field-responsive systems is the capability of non-contact stimulation and/or visualization in the body. Typically, magnetic field-responsive materials are composites consisting of a metal/metal oxide core, which can be modified by various means, e.g., by decoration with polymer chains at their surfaces, either by adsorption or covalent immobilization; by entrapment in a thermoplastic bulk polymer; by crosslinking into a polymer network structure; or by placement at the surface of larger-sized porous or sacrificial template particles, leading to hollow assemblies. Iron oxide (magnetite Fe_3_O_4_ [109] or maghemite *γ*-Fe_2_O_3_ [110]), as well as cobalt ferrite (CoFe_2_O_3_ [111]), have often been used as magnetic cores since they have high saturation magnetization and are (at low levels) typically considered to be non-toxic and suitable for access to biochemical pathways of Fe metabolization [112,113]. Superparamagnetic iron oxide nanoparticles (SPIONs) are small nanoparticles under 20 nm that exhibit superparamagnetic behavior, i.e., they have no permanent magnetization (remanence) after removal of the magnetic field.

The magneto-sensitivity of such composite systems has been employed in a number of principal concepts: (i) the accumulation and retention of freely circulating particles at a target site by a locally applied static magnetic field, e.g., for local drug release; (ii) imaging of the localization of magnetic probes targeted to disease sites for diagnostic/theranostic purposes; (iii) the induction of hyperthermia in a target tissue, typically in the context of tumors, by heating magnetic nanoparticles in an alternating magnetic field; (iv) the enhancement of passive processes such as drug diffusion by the external modification of composite properties; and (v) the recall of active predefined functions implemented in the polymer matrix. In the following, a number of examples will be provided for these concepts.

*Localized drug release.* To achieve a high local drug concentration, bioactive molecules can be covalently or physically bound to the surface or entrapped within the pores or cavities of the magnetic particle. When introduced into the body, typically via injection, the presence of a local static magnetic field results in a translational force on the polymer/drug coupled particle. This should, conceptually, lead to particle movement towards the magnet and/or trapping at the target site after distribution via blood circulation [114,115]. Ex vivo models with human umbilical arteries have been proposed as alternatives to animal studies to assess particle retention under flow conditions [116]. The particles should then release the drug via diffusion or specific mechanisms such as enzymatic reactions or via the given environmental conditions. For efficient delivery, several factors need to be considered, including physical parameters such as the number and surface chemistry of particles, the binding principles of the drugs, and the strength and duration of the magnetic field, as well as the physiological parameters of the patient such as blood flow, vascular supply, body weight, and depth to the target [117]. Because of the aggregation of virgin magnetic particles, surface chemistry has a special importance for in vivo stabilization, target selectivity, controlled circulation time, and optimal detectability [118]. Surface modification with neutral and hydrophilic compounds such as PEG, dextran or silica [119,120], or using membranes isolated from red blood cells [121], has frequently increased circulatory half-life by decreasing particle recognition by the mononuclear phagocyte system (MPS)/reticuloendothelial system (RES) due to a shielding effect/alteration of adsorbed protein corona [122]. In this way, chances increase of reaching the intended target site for vessel wall attachment, potential diffusion into the adjacent tissue, and cellular uptake. It should be emphasized that premature release during blood circulation would counteract the magnetically targeted delivery concept, while persistent binding of the drug and particle may reduce efficacy [123]. In vitro and preclinical studies [124,125,126,127], as well as clinical trials [128] (see also clinicaltrials.gov; e.g., [129,130]) have explored this concept. More recently, the interest in research has shifted towards even more complex systems including actively triggered (rather than diffusion-controlled passive) release mechanisms for (bio)pharmaceuticals, as well as the magnetically assisted accumulation of stem cells, as advanced therapy medicinal products [131].

*Imaging.* By means of magnetic resonance imaging (MRI), the localization of magnetic particles in the body can be detected, at least when nanoparticles are accumulated, using different MRI operation modes with fair resolution [132]. In this way, the soft tissue structure and pathologies can be visualized for diagnostic purposes, which can be based on commercially available or experimentally prepared magnetic probes [133]. Through a combination of magnetic particles with ligands bound to particle surfaces that match overexpressed factors [134], additional active-accumulation principles have been implemented to direct particles to the target tissue, in principle. It should be noted that despite significant improvements, the extent of accumulation at target sides is typically far from being quantitative [135]. Still, by visualizing the tissues of interest, such as small tumors, they can be subject to other treatments such as laser irradiation [136].

*Hyperthermia.* When ferromagnetic materials are subjected to alternating magnetic fields, the particles will align themselves according to the changing magnetic field. During relaxation, realignment energy is released, which allows for remote induction of local heat. Given the higher sensitivity of tumor cells to temperatures rising above ~42 °C, where enzymatic processes are diminished or inactivated, hyperthermia alone or, more relevantly, together with additional chemo- or radiotherapy, is of interest. For instance, gold radionuclides have been proposed as coating materials for magnetic nanoparticles for the combined hyperthermia/radiation therapy of small tumors [137]. Similarly, glioblastoma patients have received percutaneous beam radiotherapy immediately before or after hyperthermia [138]. The aggregation of magnetic particles due to an imperfect polymer coating can relevantly affect the ability of the particles to create heat in alternating magnetic fields [139]. While it remains a key goal to keep hyperthermia well localized [140], the unavoidable heat conduction to adjacent healthy tissue may be acceptable to a certain extent, as healthy tissue damage at 41–45 °C (the desired hyperthermia window) may be reversible.

*Magnetic field-induced drug release.* Ferrogels contain finely distributed small ferromagnetic nanoparticles with strong adhesive forces to polymer chains, through which the polymer can be subjected to macroscopic deformations such as bending or stretching in static magnetic fields via direct coupling of the magnetic and mechanical properties of the gel [141]. In addition to physical entrapment, iron nanoparticles may serve as crosslinkers in polymer networks [142]. Ferrogel membranes from polyvinyl alcohol illustrated reduced drug permeation, presumably because of pore closure when the well distributed Fe_3_O_4_ particles drive the associated polymer chains to approximate each other in the applied magnetic field [143]. This observation suggests that in vivo on-demand release control of drugs with an adjustable dose might be possible in principle. Polymersomes [144] and composite particles from hydrophobic polymers [145] showed a moderate increase in the release rates of various drugs in alternating magnetic fields in vitro, which can be attributed to softening of the polymer and enhanced drug diffusion.

**Figure 9:**
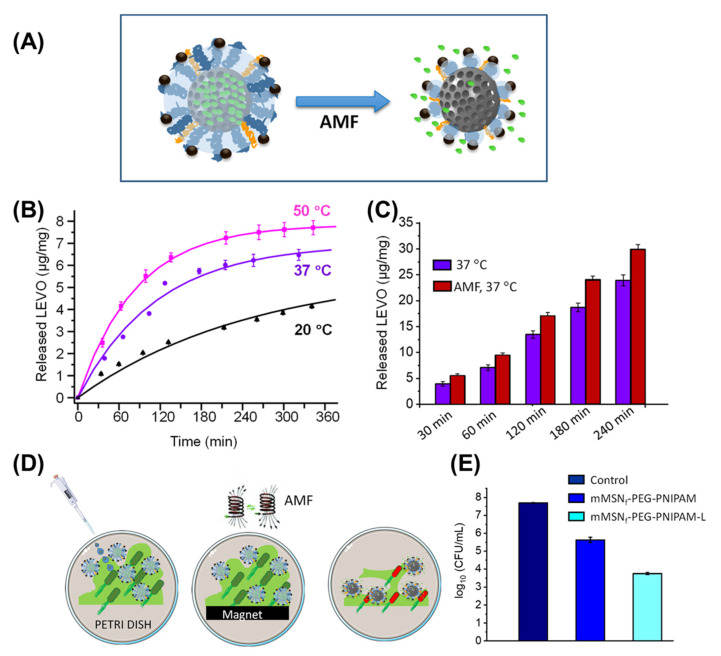
Magnetically induced drug-release system combining effects of hyperthermia and drug action. (**A**) Scheme of mesoporous silica particles with a mixed coating of PEG-SPION and PNIPAAm chains, the latter collapsing upon heating via alternating magnetic fields (AMF). (**B**) Temperature dependency of levofloxacin release in vitro. (**C**) In vitro release of levofloxacin at 37 °C with and without application of AMF. (**D**,**E**) Concept and data for the eradication of *E. coli* biofilms in vitro by hyperthermia vs. hyperthermia + levofloxacin (L) release. Reprinted from [147]; licensed under Creative Commons Attribution (CC BY) license (http://creativecommons.org/licenses/by/4.0/), © 2022 by the authors.

Through a combination of iron oxide particles with thermoresponsive polymers, reversible alterations in polymer chain conformation with consecutive shrinkage/expansion of the overall material (altered diffusivity) or of pores (pumping effect) may be realized via magnetically induced heating. Capsules composed of iron oxide nanoparticles in a matrix of thermoresponsive poloxamer with a thin silica shell (a diffusion barrier for the model drug ibuprofen) experienced a 10-fold size contraction due to the hydrophilic-to-hydrophobic transition of poloxamer upon heating, eventually leading to destruction of the capsule and on-demand drug release [146]. Another concept can be exemplified by PEG + PNIPAAm-grafted mesoporous silica particles that are filled with the antibiotic levofloxacin, which gains higher diffusivity when PNIPAAm chains collapse. Via SPIONs coupled as an outer shell to PEG, the combined bactericidal effects of hyperthermia and magnetically induced drug release were achieved on E.coli biofilms in vitro (Figure 9) [147], while quantitative biofilm eradiation and the inhibition of regrowth by residual bacteria remain key challenges of future work for this and conceptually similar systems [148]. Thermally reversible covalent drug–particle couplings, e.g., using the reversibility of Diels–Alder reactions [149], are further examples of synchronizing hyperthermia and drug release. If such on-demand release can be clinically relevant and beneficial (quantity; time frame; and comparison to direct injection) may need to be shown in specific settings in subsequent studies.

*Complex magneto-responsive switching functions.* The softening of polymer chains via indirect heating has also been the basis for magnetically induced shape-memory materials, as conceptually introduced in Section 2.1. In addition to the homogeneous distribution of magnetic particles in hydrogels or hydrophobic elastomers, anisotropic composites, the rearrangement of magnetic particles, and/or remagnetization are also explored for the complex movements and reprogrammability of SMP. SMP-derived actuator materials, which can undergo repeated cycles of movement as free-standing devices without intermediate programming, have been realized by combining actuation domains with a cage of other domains, directing the melt-induced contraction (MIC) and crystallization-induced elongation (CIE) of the actuation domains [150]. Such materials have been a major breakthrough in SMP technology and have paved the way to current concepts of biomedical soft robots/micromachines [151]. It remains to be shown if, beyond inducible shape switching for implant anchoring or microsurgery, drug release can also be implemented as a beneficial feature—again, with critical consideration of the advantage over separate (peroral or parenteral) drug administration.

Overall, since the magnetic gradient decreases with the distance to the target, the strength of the external field that can be applied and is compatible with the clinical setting is important to evaluate. This is evidenced by the development of alternating magnetic field applicators for hyperthermia, with a few systems being in routine clinical use for selected tumors (e.g., in Germany; designed by the company Magforce). In order to retain a magnetic carrier–drug vehicle at a specific location, the applied static field needs to have a relatively strong gradient, allowing it to overcome shear forces in the blood stream under dynamic conditions. Retention characteristics may thus be altered when changing from small animals with near-surface targets to larger animals and humans. Furthermore, the always-existing heat loss to sample environments (studies are often performed in a dry state in vitro) may not necessarily correspond to fluid cooling under dynamic flow conditions in vivo. Another limitation relates to the small size of NPs, a prerequisite for superparamagnetism, and their colloidal stability in fluids, which may be impeded by protein adsorption that is not fully solved by existing coating strategies [152].

### 2.4. Ultrasound-Responsive Materials

Ultrasound waves are a well-accepted medical imaging technique and may, in pharmaceutical use, be an attractive stimulus because of the simplicity of application in vivo with spatial and temporal control. In contrast to light as a trigger, ultrasound can easily penetrate deep into the body, where ultrasound waves can cause cavitation, create heat, induce acoustic streaming (localized shear stress; temporary cell membrane opening), and/or generate reactive oxygen species (ROSs) via/for cell damage, etc. [153]. Ultrasound has been suggested to allow on-demand drug release to induce membrane permeability (e.g., blood–brain barrier permeation; enhanced cellular uptake), to enable image-guided therapy (theranostics), and to indirectly stimulate heat responses in thermosensitive materials. It should be noted that in addition to the direct application of ultrasound for sonography, the generation of ultrasound waves for imaging is also possible via the photoacoustic effect, as shown for dye-loaded PLGA nanoparticles under the application of near-infrared laser pulses [154].

*Burst of bubbles.* Micro-/nanobubbles contain air or water-insoluble gases such as perfluorocarbons encapsulated in shell structures from polymers or other materials. They can enhance ultrasonic signals, thus serving as clinically relevant ultrasonic contrast agents (e.g., Optison ^TM^, Definity ^®^, SonoVue ^®^), and are, at the same time, extensively studied for ultrasonically triggered drug release. To generate ultrasound waves, two types of technology are mainly utilized: low-intensity focused ultrasound (LIFU; ~20–100 kHz) and high-intensity focused ultrasound (HIFU; ~1 MHz), which can cause stable cavitation and inertial cavitation, respectively. HIFU at a suitable excitation pressure is often applied for microbubble destruction (rather than imaging), as HIFU typically matches their resonance frequency, allowing for maximum oscillation (depending on the bubble size, size distribution width, shell mechanics, etc.) [155]. Nonlinear and nonspherical oscillations of bubbles are associated with higher levels of microstreaming, which—similarly to shock waves during microbubble implosion—provide shear stress on adjacent cells, allowing for (ideally temporal) cell membrane disruption (sonoporation). This phenomenon can support the passage of drugs to the cytosol, presumably working best for water-soluble molecules that have a relatively small molecular weight and high diffusion coefficients [155]. Microbubbles, which were formed by the coalescence of nanobubbles at the target site after extravasation, provided a strong and long-lasting tumor contrast in ultrasonography and, in combination with DOX-loaded polymeric micelles, may be a considerable concept for breast tumor treatment, as suggested by in vivo studies with targeting and retention at the tumor site [156]. Similar results were found for intravenously injected curcumin/PFP-loaded nanobubbles from PEG-*b*-PCL, which were highly echogenic in mice, probably due to coalescence, and showed better suppression of tumor growth when ultrasound was applied [157]. In addition to gas-filled bubbles, similar systems have been studied that form a gas-filled core from a liquid via phase transition [158].

*Disturbance of chain organization in hydrated vesicles/particles.* The ultrasound-induced disturbance of polymer (self-)organization has been the basis for investigating micellar systems, for instance, early works on DOX release from poloxamer in a zero-order fashion upon sonication with first-order re-encapsulation when the ultrasound treatment is stopped [159,160]. Another study indicated that, in addition to physical disruption of block copolymer (BCP) micelles and the concomitant release of encapsulated Nile red dye, chemical alteration such as hydrolysis may also occur under the selected conditions for some block copolymers, but not for PEG-*b*-PMMA micelles [161]. A reduction in undesired premature drug leakage from hydrated vesicles has been suggested to occur via dual sensitivity, i.e., setting higher requirements to be overcome for the onset of drug diffusion. For instance, dendritic/hyperbranched nanoparticles should only release their drug payload when drug cleavage from pH-sensitive imine bonds occurs under intracellular conditions (pH < 5), and ultrasound induces drug expulsion from the outer layer of the dendrimer [162]. In principle, further stimuli-sensitive units can also be implemented, as exemplified in particles comprising iron-oxide cores, polymer coatings with pH-dependent swellability, and redox-sensitive disulfide bonds, which furthermore showed an enhancement of drug release via ultrasound [163]. While sensitivity to multiple triggers can scientifically be interesting, a clinical translation seems more probable for stimuli-responsive systems with a lower number of variable parameters.

*ROS production.* Along with the increasing interest in sonodynamic tumor therapy, which involves the administration of sensitizing components such as hematoporphyrins and various experimental dyes to support the formation of ROSs in the vicinity of tumor cells, nanocarriers are of interest as sensitizers [164]. The concept of using metal–organic-framework-derived nanostructures containing porphyrin, such as macrocycles, which could efficiently generate ROSs as active agents under experimental ultrasound application in vitro, has recently been highly recognized in the scientific community [165].

**Figure 10:**
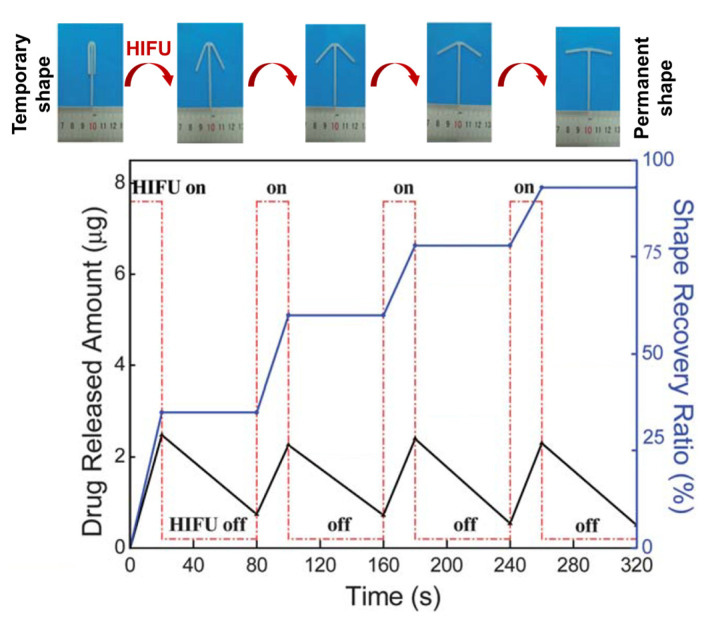
Stepwise shape-recovery and release of copper sulfate as a model compound from temperature-sensitive amorphous SMP networks from poly[(methyl methacrylate)-*co*-(butyl acrylate)]. High-intensity focused ultrasound (HIFU) was applied as non-contact stimulus that indirectly creates heat. Modified with permission of Royal Society of Chemistry, from [73]; permission conveyed through Copyright Clearance Center, Inc.

*Ultrasound-induced heating.* One other major effect linked to ultrasound is mechanical friction generated by the acoustic energy adsorbed by polymer matrices and tissues, causing local heat. HIFU is used for ablation therapy (rapid tissue heating to >60 °C; maintained for >1 s) as well as hyperthermia in tumor combination therapy (e.g., 44 °C for 60 min) [166]. Such ultrasound-induced heat can also mediate the diffusion of drugs from reservoir- or matrix-type carrier systems. Poly(*N*-(2-hydroxypropyl)-methacrylamide mono/dilactate)-grafted liposomes, in which the thermosensitive chains coil and interact with the lipid bilayer above the polymer’s LCST, showed strongly enhanced DOX release by HIFU [167]. In another study with a temperature-sensitive acrylate-based SMP network (Figure 10), HIFU allowed the system to heat up to 100 °C in an aqueous environment and reversibly enhanced the diffusivity and release of copper sulfate as a model component [73]. Considering the harsh conditions, this treatment may conflict with the aim of preserving viable tissues at the application site at <43 °C, and thus, requires further development.

Overall, it is obvious that ultrasound-triggered release systems are the subject of extensive academic research. It may be important to consider clinically applicable ultrasonic conditions (which differ from those of ultrasound water baths/ultrasound homogenizers available in most laboratories) already during exploratory studies in order to better judge the opportunities arising from the proposed technologies.

## 3. Responsiveness to In Vivo Stimuli

### 3.1. pH- and/or Ion-Responsive Materials

**Figure 11:**
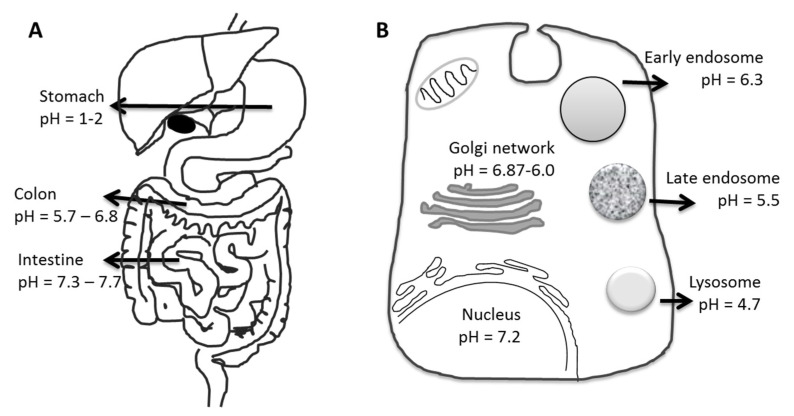
Schematic illustration of pH levels in the body (**A**) in the gastrointestinal tract and (**B**) at the cell level (a prototypical mammalian cell).

The concept of using pH-responsive materials as drug carriers relies on variations in physiological pH at various body sites, from organ to tissue and even to cell levels in normal as well as pathological conditions. The gastrointestinal tract (GIT) is an important example for the application of pH-sensitive polymers since the pH value varies along the GIT from stomach to colon (Figure 11). The pH profile of pathological tissues, such as the site of infections, inflammation, or tumors, is also significantly different from that of normal tissue [168]. Cellular components such as the cytoplasm, endosomes, lysosomes, Golgi bodies, and nuclei are known to maintain their own characteristic pH values in all tissues (Figure 11B) [168]. Polymer particles can be taken up into tissue cells via fluid-phase pinocytosis or receptor-mediated endocytosis. The formed intracellular vesicles are cellular compartments that experience pH changes from 6.3 to 4.7 from the early endosome to the lysosomes, thus giving a distinct change in proton concentration to affect the properties or structure of polymers/carriers.

Two major approaches have been used to trigger drug release via a pH drop: (i) ionizable groups that introduce structural changes or destabilization in self-assembled vesicles composed, e.g., of block copolymers, and (ii) conjugation chemistry with pH-labile chemical bonds (drug–polymer or in-between polymer components that entrap the drug) [169,170].

Polymers with ionizable groups in their backbone can form polyelectrolytes in aqueous media. As the environmental pH changes, the ionization degree of these groups is dramatically altered depending on their p*K*a. Weak polyacids accept protons to become uncharged at a low pH and release protons at a neutral and high pH [171], while polybases gain positive charges when shifting to acidic conditions. Such a rapid change in the net charge of the pendant groups causes an alternation in the hydrodynamic volume of the polymer chains, e.g., from a collapsed state to an expanded state due to ionic repulsion of the chains, as well as osmotic pressure effects from the mobile counter ions [172].

Typical examples of cationic polyelectrolytes are tertiary aliphatic amines, morpholino, pyrrolidine, piperazine, and imidazole groups such as in poly(*N*,*N*′-dimethylaminoethyl methacrylate) (PDMAEMA), poly(acryloylmorpholine) (PAM), poly(ethylenimine) (PEI), and poly(amidoamine) [173]. In addition to linear polymers, branched, hyperbranched, and dendritic polyelectrolytes are also investigated. Poly(vinyl pyridine) (PVP) undergoes a phase transition at pH < 5 as a consequence of the protonation of pyridine groups [174,175] and imidazole, as a weak base in poly(vinyl imidazole) (PVI), is responsible for the protonation of this polymer in acidic solutions [176]. Poly(propylene imine) (PPI) dendrimers for applications in pH-responsive controlled-release systems can provide multiple functional groups for reversible compound binding due to their dendrimeric structure [177,178]. In addition to synthetic polymers, biopolymers such as chitosan also revealed a potential for pH-controlled release, as studied in gastric pH medium using chitosan hydrogels blended with polyvinyl pyrrolidone and loaded with cefixime as an antibiotic drug [179]. However, the sharpness of the pH transitions of biopolymers was rather poor compared to polycarbonate materials containing tertiary amines as side groups, where the apparent p*K*a was inversely correlated with amine hydrophobicity and could be tailored (p*K*a 5.2 to 7.7) to sharp transitions in very narrow pH windows; this can be relevant for the intracellular pH steps [180].

Polymers sensitive to anionic pH are mostly based on carboxylic, sulfonic, phosphoric and boronic acids or on sulfonamides such as PAAc [181], poly(methacrylic acid) (PMAAc), and poly(2-acrylamido-2-methylpropane sulfonic acid) (PAMPS). The p*K*_a_ of sulfonamide-based polyelectrolytes can be systematically varied from 3 to 11 depending on the electro-withdrawing nature of the substituent of the nitrogen [182]. An early polymer system that enabled precise control of the pH of conformational transition was designed based on the Hammett equation, eventually leading to 4-amino–*N*-(4,6-dimethyl-2-pyrimidinyl)benzene sulfonamide–*N*,*N*-dimethylacrylamide copolymers reversibly switching, e.g., at pH 7.0 [183]. Compared to weakly acidic conditions (physiological skin pH), crosslinked hydrogel nanofibrous mats based on poly(acrylamide-co-acrylic acid) showed excessive swelling at a neutral or slightly basic pH (chronic wound conditions), as well as a slightly faster in vitro release of amoxicillin, making them candidates for exudate absorption and local infection therapy as active wound dressings [184].

Beyond charged groups, another important design parameter is the presence of hydrophobic moieties added to the polymer backbone. Hydrophobic groups may allow a more compact conformation to be obtained at the uncharged state, resulting in a more distinct phase transition depending on the number, nature, and distribution of the groups on the chain. For instance, as an example of a polyacid, poly(methacrylic acid) (PMAAc) can undergo more extensive hydrophobic interactions than PAAc in its protonated state based on its methyl groups, allowingPMAAc chains to adopt a compact conformation and macroscopically presenting an abrupt volume phase transition upon deprotonation compared to the relatively more continuous volume phase transition of PAAc, which lacks methyl side groups. Hydrophobic comonomers can also affect the switching pH, which increased with the content of hydrophobic octyl acrylate in poly(acrylic acid–*co*–octyl acrylate) copolymers [171]. Similarly, the pH of the transition of poly(methacrylic acid–*co*–ethacrylic acid) (P(MAAc–*co*–EAAc)) changed from pH 5.6 to 6.6 when the EAAc content was altered from 49 to 100 mol% [185].

Based on such polymers, various pH-responsive delivery systems, including homopolymers, block copolymers, micro-/nanogels, hyper-branched structures, and vesicles, have been suggested for the efficient intracellular delivery of macromolecules to distinct sub-cellular compartments. Hollow nanocontainers inspired by virion particles were synthesized from PAAc via vesicular polymerization or emulsion polymerization (using core-shell latex particles) and demonstrated pH-induced dissociation of carboxylate groups, resulting in swelling and increased permeability of the capsule shell with increasing pH [186]. Poly(*N*-isopropylacrylamide-*co*-vinylimidazole)-based particles protected the entrapped DOX under simulated bloodstream conditions (pH = 7.4; 36 °C), while the drug was nearly completely released due to the solubility of the polymer in slightly acidic environments, i.e., the pH values found in cell compartments [187]. The organization of polymer chains into superstructures, such as helices in polypeptides, can contribute to stabilizing polymeric assemblies, despite the presence of good solvents. For giant vesicles from amphiphilic block copolypeptides, the protonation of some lysine residues at a low pH and an associated drop in hydrophobicity led to a helix-to-coil transition of the block copolymer, dissociation of the vesicle, and the release of an encapsulated probe [188].

**Figure 12:**
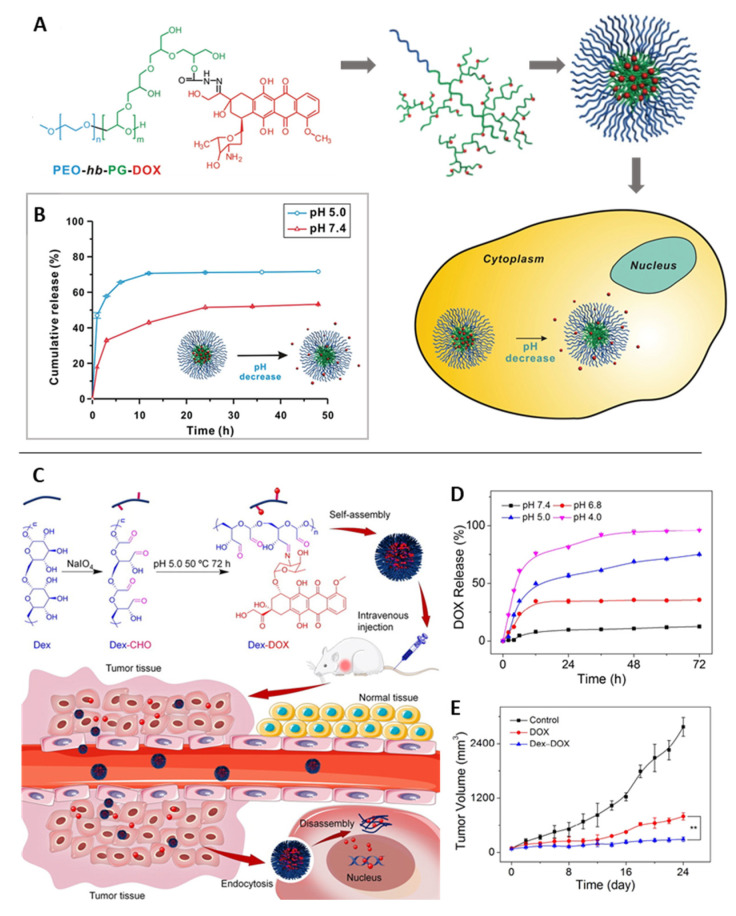
pH-triggered drug-delivery systems for doxorubicin (DOX) based on cleavable bonds in different positions based on (**A**,**B**) hydrazone and (**C**,**E**) imine links. (**A**) Scheme of DOX coupling to hyperbranched double-hydrophilic block copolymer (PEO-*hb*-PG-DOX), its micellar assembly, and intracellular drug release. (**B**) DOX release profiles under different pH conditions. Reprinted with permission from [190]. Copyright 2012, American Chemical Society. (**C**) Scheme of DOX coupling to dextran (dex) and proposed mode of action of ~23 nm-sized particulate aggregates. (**D**) pH-dependent DOX release in vitro. (**E**) Tumor volume of B16F10 melanoma-grafted BALB/c mice treated with free DOX, DOX-dextran nanoparticles, or PBS as control. Reprinted from [192]. Copyright 2017, with permission from Elsevier.

As introduced above, bond cleavage is another general principle that enables pH sensitivity, for which several different types of linkages can be facilitated in principle [189]. For drug release via the cleavage of drug-polymer conjugates, acid-labile hydrazone linkages are very commonly employed. For instance, DOX was coupled to block copolymers of hyper-branched polyglycerols and poly(ethylene oxide) (PEO-*hb*-PG) [190]. The conjugation of DOX to the various binding sites of the hyperbranched polyglycerol altered its hydrophilicity, leading to spontaneous self-assembly as micellar aggregates. Upon exposure to an acidic condition, the DOX in vitro release was accelerated, particularly at early time points (Figure 12A,B). Acid-labile imine bonds have also been regularly used as pH-responsive linkages, e.g., in a “nanococktail” for the codelivery of the anticancer drugs (i) epirubicin complexed with phospholipids and (ii) methotrexate (MTX) linked to a lipid-PEG conjugate via a covalent imine bond. These components were combined via self-assembly to form an MTX-targeted nanoparticle that allowed the combination of targeting tumor cells and the release of drugs at an acidic pH [191]. DOX-coupled dextran aggregated into very small-sized nanoparticles, from which DOX was released at gradually increasing rates upon pH-shifts from 7.4 to 4.0 via imine bond cleavage, with mouse studies showing more effective tumor volume suppression compared to free DOX due to reduced renal excretion of the DOX-dextran nanoparticles [192] (Figure 12C,E).

**Figure 13:**
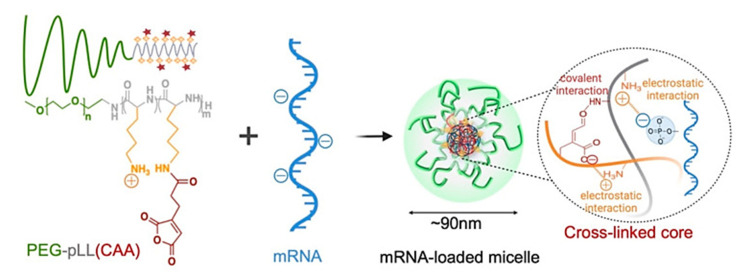
Illustrative scheme of mRNA-loaded micelle sensitive to environmental pH due to cleavable amide bonds. Vehicle formation includes covalent crosslinking of CAA (*cis*-aconitic anhydride) and pLL (poly(L-lysine)) in micelle cores and electrostatic interaction between mRNA and pLL. Reprinted from [196]; licensed under Creative Commons Attribution (CC BY) license (http://creativecommons.org/licenses/by/4.0/), © 2022 by the authors.

pH-sensitive polymers have also gained attention in gene delivery, where the anionic nature of (deoxy)ribonucleic acids motivated the use of reversibly charged carriers. Due to their size and charge under physiological conditions, the passive diffusion of DNA/small interfering RNA (siRNA) into a cell is inhibited. Therefore, cationic polymers are usually used to compensate the charge of nucleic acids and condense them to polyplex nanoparticles of, e.g., ∼100 nm in size. These cationic polymers are then deprotonated within the endosomes, which triggers the disruption of endosome membranes and payload release to the cytosol, according to the proton sponge theory. This rupture should occur before fusion with lysosomes that contain hydrolytic enzymes and might affect DNA/RNA integrity [193]. Through the alteration of polymer structures, the protonation/deprotonation pattern, and thus, the response in a specific type of cellular compartment can be adjusted. Examples include non-toxic DMAEMA/HEMA nanoparticles, which could support transfection as they are attributed to particle uptake; endosomal swelling of DMAEMA/HEMA nanoparticles; and subsequent delivery of the DNA via intracellular diffusion [194]. Protonated Schiff-base linkages and imidazole moieties in functionalized chitosan were also capable of providing suitable interaction with DNA for polyplex formation, which was reversible at decreased pH, resulting in DNA release [195]. Poly(ethylene glycol)-*b*-poly(L-lysine)-based micelles were explored for binding mRNA in their crosslinked pH-responsive core (amide bonds between ε-amine of poly(L-lysine) and *cis*-aconitic anhydride (CAA) coupled to the main chain) via multivalent ionic complexation, suggesting complex stability at physiological pH (pH 7.4) and complete release of the mRNA via complex dissociation at endosomal pH (pH 5.5–4.5) (Figure 13) [196].

**Figure 14:**
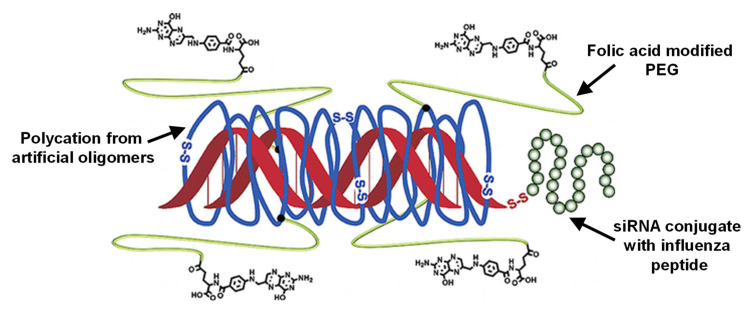
Designer polyplexes comprising polycations built from charged artificial oligomers with efficient siRNA-binding capability, and grafting with PEG for shielding and with folate for receptor-mediated cellular uptake. siRNA can be bound in polyplexes of defined size via ionic interaction and can be used as conjugate with an endosomolytic peptide (herein, influenza peptide Inf7). Adapted with permission from [199], Copyright © 2022 American Chemical Society.

To enable endosomal escape, recent studies have also facilitated endosomolytic peptides, which are incorporated into polyplexes. Examples include the gene-silencing activity of siRNA polyplexes coated with the endosomolytic peptide diNF-7, which also preserve their silencing activity in the presence of serum proteins [197]. Furthermore, grafting polylysine with (i) PEG to induce a shielding function during circulation, (ii) melittin peptide for endosomolysis, and (iii) siRNA via a redox-sensitive disulfide bond for intracellular release was suggested to overcome the undesired extracellular instability in siRNA polyplexes [198]. This concept can be further expanded by adding targeting moieties to direct tissue distribution and the use of designer polycations for efficient siRNA binding (Figure 14) [199].

In some cases, the two mechanisms of pH sensitivity, i.e., ionizable groups and bond-cleavage at low pH, were combined. Examples are polyacylhydrazone polymers that combine neutral PEO groups and cationic moieties for oligonucleotide binding; the latter is able to effectively complex dsDNA in biological media and degrade via acid-catalyzed hydrolysis at pH 5.0–7.0, thereby possibly promoting endosomal escape [200]. It should be noted that beyond endosomal escape to the cytosol, trafficking of the payload into the nucleus is a further essential step for DNA delivery. Furthermore, it should be considered that the interaction of preformed polymer vesicles and nucleic acids as relatively large molecules can cause substantial rearrangement of the polymer, potentially forming nanoparticulate aggregates that no longer possess micelle-like chain organization (despite being presented as such in conceptual figures).

**Figure 15:**
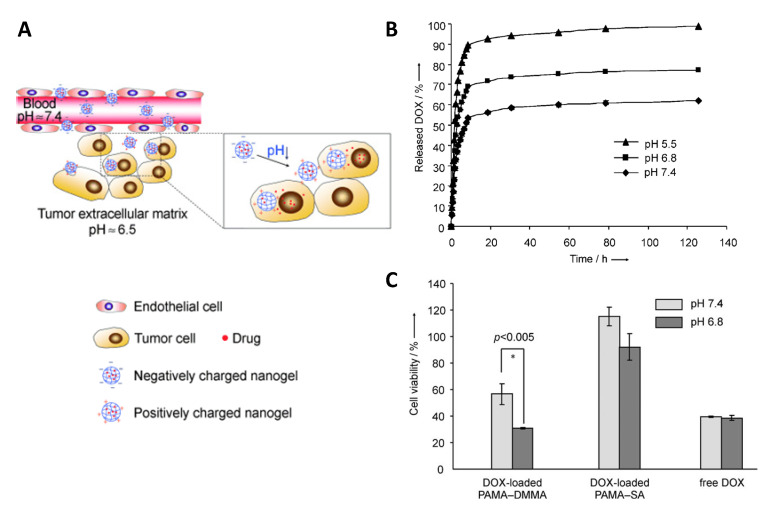
pH-sensitive nanogels from poly(2-aminoethyl methacrylate hydrochloride)-2,4-dimethylmaleic anhydride (PAMA–DMMA) with charge conversion. (**A**) Scheme of proposed action of PAMA–DMMA nanogels that are negatively charged in the blood, leak into tumor sites due to the EPR effect, and experience charge conversion to positive charge in the acidic tumor environment, which may enhance cellular internalization. (**B**) pH-dependent cumulative DOX release profiles from the PAMA–DMMA nanogels in vitro. (**C**) Cell viability of MDA-MB-435s cells after incubation with DOX-loaded PAMA–DMMA and free DOX at the same DOX concentration. Reprinted with permission from [203], 2010 Wiley-VCH Verlag GmbH & Co. KGaA, Weinheim.

Metabolic differences between healthy and tumor tissues, specifically lactate-based acidosis in tumors (pH 6.0–6.5) [201], motivated researchers to use low pH as an in vivo stimulus for cancer targeting. A combinational drug therapy was addressed using an injectable pH-responsive peptide hydrogel, which is based on β-sheet formation and peptide assembly to nanofibers at physiological pH, which are disrupted in slightly acidic conditions. When coencapsulating the antitumor drugs gemcitabine (GEM) and paclitaxel (PTX) for injection in the vicinity of tumors, only PTX release (7 days: 97% at pH 5.8, 39% at pH 7.4) but not GEM release (3 days: 100% at pH 5.8, 100% at pH 7.4) was pH dependent in vitro, probably due to the already high diffusivity of hydrophilic GEM [202]. A nanogel of poly(2-aminoethyl methacrylate hydrochloride) (PAMA) cross-linked with poly(ethyl-ene glycol) (PEG) diacrylate was surface-modified at ~63% of the amine groups with 2,4-dimethylmaleic anhydride (DMMA) to introduce a negative net charge (charge dominated by deprotonated carboxyl groups at pH 7.4). Under slightly acidic conditions, the protonation of carboxyl groups caused a transition to a positive net charge by free amine moieties, as desired to enhance the cellular uptake of nanogels, promoted cargo release, and more efficiently killed cancer cells in vitro (Figure 15) [203].

These examples indicate that polymers with pH-responsiveness may be applied for the design of advanced drug carriers for on-demand drug delivery, with high relevance for gene delivery, as well. Rapid progress in the area of polymer synthesis and controlled polymerization techniques stimulated the design of sophisticated multifunctional carriers with well-defined compositions. Recent experimental concepts have addressed the challenge of equipping the carriers with conflicting functions such as elevated circulation time and enhanced biological interaction such as for cellular uptake or endosomal escape. It appears that a combinatory approach is needed involving several functional materials, which may make it laborious to prove the contribution and safety of each component for therapeutic application. As explained with several examples, polymeric systems responsive to tumoral or intracellular pH have been extensively studied in vitro, and in some cases, confirmed in animal trials; however, the validation of their safety and efficacy in humans is so far not sufficient and may be the subject of clinical studies.

### 3.2. Enzyme-Responsive Materials

The selective use of the catalytic actions of enzymes has recently gained increasing attention for drug delivery. Enzymes function under mild conditions and play key roles in healthy and diseased sites; therefore, enzyme-responsive materials operable under mild conditions in vivo may offer spatiotemporal control of drug-release systems. Such polymers typically possess an enzyme-sensitive moiety along the main chain or have suitable side groups, possibly combined with a second unit to account for the typically noncovalent interactions associated with a triggered structural transition [204]. Enzymatic action on these polymers is typically not reversible but causes structural changes by following one of three main principles: (i) the induced self-assembly of polymers, (ii) the disintegration and structural reorganization of supramolecular assemblies and nanoparticles, or (iii) the formation or cleavage of covalent networks, leading to sol–gel or gel–sol transitions [204,205].

Fundamental research has illustrated different principles on how enzymatic cleavage or polymerization reactions (e.g., side chains, main changes, crosslinks in networks, surface coatings) could modify (co)polymer properties and, in consequence, the state of carrier systems. Vinyl monomers bearing a cleavable enzymatic substrate as a side chain were polymerized to obtain water-soluble double-hydrophilic block copolymers, which become amphiphilic and self-assemble into a micelle structure upon enzymatic cleavage of the side groups [206]. Phosphorylase b was used to extend the oligosaccharide block of an amylose-based surfactant via enzymatic phosphorylation, which led to structural rearrangement from a micellar structure to form vesicles when surpassing a certain length [207]. For poly(acrylamide-*co*-dextran methacrylate) nanogels comprising dextran crosslinks in addition to the acrylate-based network structure, the comparably fast enzymatic degradation of dextran by dextranase resulted in a substantial increase in the swelling degree, which might be facilitated to enable the release of a payload [208]. In a recent study, knowledge of the overexpression of hyaluronidase (HAase) was suggested for precise atherosclerosis (AS) treatment through the design of enzyme-responsive and macrophage-targeting mesoporous silica nanoparticles (MSNs) coated with hyaluronic acid (HA); the HA should be cleaved off the particle surface in the target tissue, allowing for higher simvastatin release from the particle core in atherosclerotic plaques [209]. The potential of HA to interact with CD44 receptors has also been repeatedly investigated for homing NPs to tumor tissues, where the removal of HA from the particle surface using extracellular HAase should support subsequent tumor penetration of the carrier [210].

**Figure 16:**
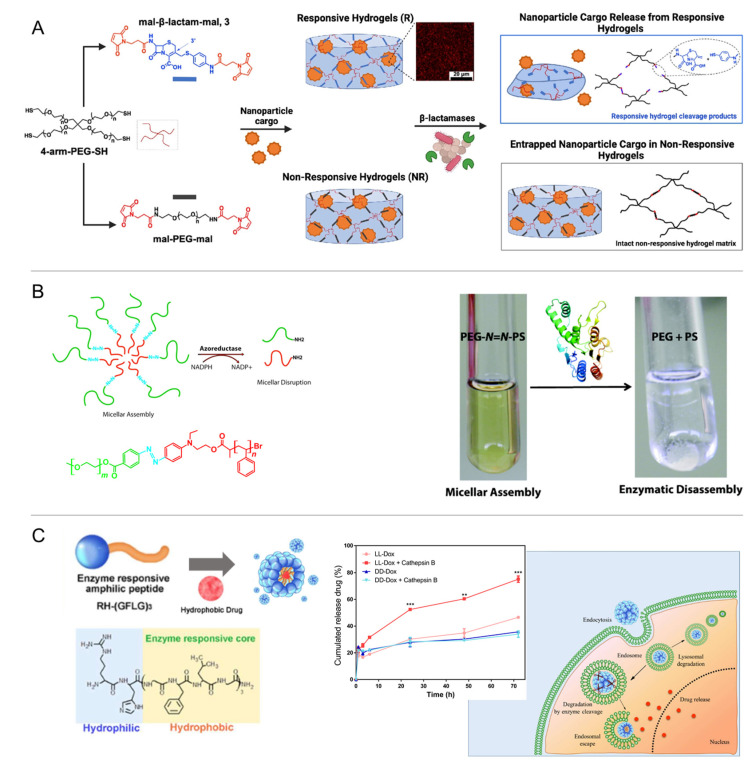
Enzymatic cleavage for disassembly of carrier systems. (**A**) Cleavage of cephalosporine junction by bacterial β-lactamase for hydrogel destruction. Reprinted with permission from [215]. Copyright 2022, American Chemical Society. (**B**) Cleavage of azobenzene-linked poly(ethylene glycol)-*b*-poly(styrene) (PEG-N=N-PS) amphiphilic copolymer causing micellar disassembly by azoreductase in the presence of NADPH. Reprinted with permission from [219]. Copyright 2013, American Chemical Society. (**C**) Cleavable amphiphilic peptide (Arg-His-(Gly-Phe-Lue-Gly)_3_ (RH-(GFLG)_3_) with GFLG sequences sensitive to intracellular cathepsin B. Reprinted from [228]; licensed under Creative Commons Attribution (CC BY) license (http://creativecommons.org/licenses/by/4.0/), © 2022 by the authors.

Even though the number of known enzyme/substrate pairs is very high, to date, the employed types of catalysis reactions are limited. Enzyme-responsive carriers mainly rely on redox reactions or bond formation/cleavage, such as the hydrolysis of esters or short peptide sequences, by, e.g., proteases, kinases, and phosphatases. Enzymatic substrates constructed into the carrier systems are often biopolymers, i.e., peptides, polysaccharides, or nucleic acids. For instance, cancer-associated proteases (CAPs), including urokinase plasminogen activator (uPA) and membrane-type matrix metalloproteinase (MMP), catalyze the hydrolysis reaction of caged liposomes, which disintegrate via osmotic swelling of the liposome core once the consensus sequence peptide of µPA (Ser-Gly-Arg-Ser-Ala) is cleaved in the polymer cage [211]. DNA-decorated nanocapsules, which were constructed via enzymatic DNA coupling onto shell-crosslinked micelles, disassembled in the presence of esterases and released the cargo of their hydrophobic cores [212]. Similarly, the HAase-catalyzed hydrolysis of disaccharide links of HA segments triggered the swelling/disintegration of methacrylate-crosslinked nanogels and an enhanced release of cytostatic drugs [213]. The specificity of DNA recognition has been facilitated to release intercalated doxorubicin from DNA nanoprisms via the rate-limited cleavage of oligonucleotides [214]. In addition to the poly/oligomeric substrates of an enzymatic attack, small molecules such as cephalosporin can also serve as a cleavable backbone unit integrated into polymer networks, in this case, facilitating bacteria-mediated hydrogel destruction by secreting β-lactamase, e.g., in wounds (Figure 16A) [215].

Considering the variety and extensive availability of enzymes in the gastrointestinal tract, the physiological microbial flora may be employed to alter enzyme-responsive poly-mers, e.g., for colon delivery. Enzymes of interest included microbial dextranase for degradation of dextran-based hydrogels with diisocyanate crosslinks [216] and azoreductase to cleave azoaromatic bonds and release proteins entrapped in hydrogels [216,217,218]. A micellar system from an amphiphilic poly(ethylene glycol)/polystyrene diblock copolymer (PEG/PS) comprising an azobenzene linkage was driven to disassembly via cleavage of the azo-based copolymer linkage (Figure 16B) [219]. A lysosome-responsive hydrogel based on methacrylated carboxymethyl chitosan (MA-CMCS), efficiently encapsulating hydrophobic imatinib and sodium deoxycholate, was designed to enable intestinal enzyme-responsive release and to open epithelial tight junction for the enhanced treatment of colon cancer [220].

Many researchers have also studied polymer therapeutics with enzyme responsiveness for anticancer therapy, e.g., copolymer conjugates based on water-soluble *N*-(2-hydroxypropyl) methacrylamide (HPMA) with peptide spacers that are ideally stable in blood circulation but are attacked intracellularly, e.g., by cathepsin B in the lysosomes [221,222,223,224]. A number of anticancer drugs such as DOX, geldanamycin (GDM), mesochlorin e6 (Mec6), methotrexate (MTX), 1,5-diazaanthraquinones (DAQs), and platinate were attached to the side chain of an HPMA copolymer, and several of these HPMA–drug conjugates have entered Phase I/II clinical trials [225,226]. Other polymers employed for drug conjugates include PEGs of linear or branched architecture, assuming that they form micelles after conjugation with a hydrophobic unit, e.g., DOX, via different peptide linkers and show enhanced circulation time and a reduction in unwanted exposure of heart tissue to limit systemic toxicity. Experiments in mice bearing either a subcutaneous B16F10 tumor or an intraperitoneal L1210 tumor confirmed the activity of selected PEG−DOX conjugates and stressed the EPR theory as a means to enhance tumor targeting [227].

In addition to intracellular enzymes, the catalytic capabilities of secreted enzymes were evaluated for controlled delivery. This includes plasmin, a serine protease in the blood, which was used to cleave peptide crosslinkers in ~35 nm acrylamide-based nanocapsules; this resulted in the sustained in vitro release of intact protein depending on the concentration ratio of cleavable/non-cleavable crosslinker [229]. Matrix metalloproteinase-2 (MMP2), as it is overexpressed in some tumors, was suggested to attack the octapeptide sequences (Gly–Pro–Val–Gly–Leu–Ile–Gly–Lys) of a thermogelling poloxamer-based multiblock copolymer, indicating a bioresponsive release of PTX from micelles in vitro depending on the enzyme concentration [230]. Lipases, often used as model enzymes for degradation studies, can adopt a dual role as catalysts for polymer synthesis in non-aqueous phases and as hydrolyzers of the ester bonds, leading to bolus release of the payload in aqueous environments [231]. The cleavage of poly(alanine-*co*-phenylalanine) [PAF] segments of a thermogelling PEG-*b*-PAF copolymer system by mammalian proteolytic enzymes disturbed their β-sheet polypeptide conformation and gel integrity, eventually supporting the sustained release of insulin for blood glucose control in vivo [232]. In a recent study, nanoparticles from the amphiphilic peptide Arg-His-(Gly-Phe-Lue-Gly)_3_ (RH-(GFLG)_3_) were reported, where the hydrophobic GLFG sequence is sensitive to cathepsin B in simulated intracellular (endosomal) conditions, allowing for the faster release of DOX (Figure 16C) [228].

Despite such advanced and intelligent platforms, compared to other responsive smart materials, the clinical translation of enzyme-responsive polymeric structures will ideally require very specific enzymes and corresponding enzyme-labile moieties that would exclusively be present at the target site of the pathophysiological condition—a great (potentially too great) challenge since biomolecules are seldom found to be absolutely absent and/or are rarely present only at specific sites of the body. Additionally, inertness of the material to modifications by other components of the biological environment may not be given in many cases. Furthermore, for enzyme-triggered on-demand release, substantial differences in the drug-diffusion kinetics in the carrier material with and without enzymes are needed in order to allow efficient drug entrapment over extended time periods without leakage. On the other hand, for tight network structures, steric effects may impede the access of the enzyme into the hydrogel, and thus, its interaction with the substrate motif.

### 3.3. Systems Switching in Response to Small-Molecule Stimuli—From Physiological Markers to Danger Signals

The response of drug-carrier systems to in vivo stimuli may lead to self-regulated release systems that adapt release rates to the concentration of marker molecules, e.g., within circadian cycles. However, a critical point is to identify markers that represent a surrogate of the present disease status and can undergo highly specific interaction with a ‘sensor’ component within the polymer to induce drug release; this is typically achieved via the alteration of distinct material properties, such as the swelling/deswelling of hydrogels via the formation/disturbance of physical interactions or via chemical reactions.

**Figure 17:**
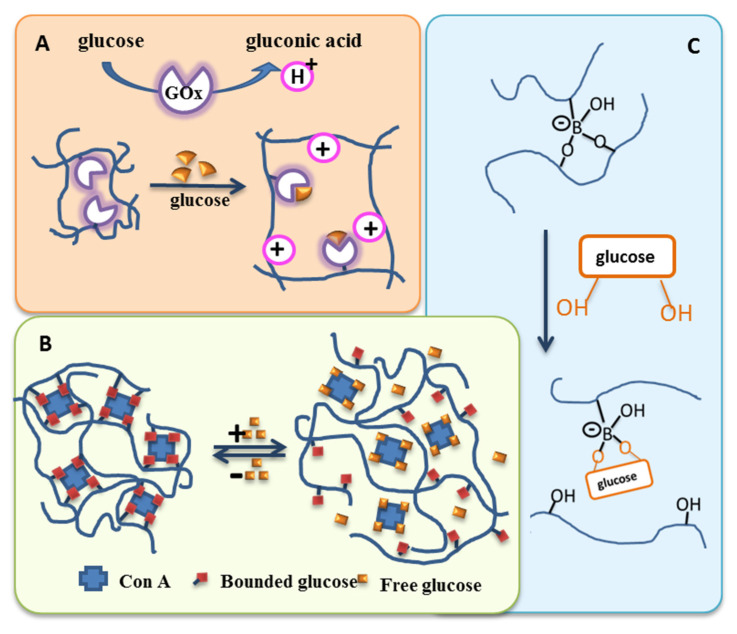
Main principles of glucose sensing for on-demand insulin release via enhanced diffusivity out of hydrogel matrices. (**A**) pH-responsive polymer hydrogels including glucose oxidase (GOx) as sensor, which oxidizes glucose to gluconic acid, causing a pH change, and thus, swelling of the network. (**B**) Competitive binding of free glucose to Con A leads to network loosening. (**C**) Covalent binding of glucose to boronic acid leads to netpoint destruction and gel–sol phase transition. Various systems based on these principles have been reported, some of which include a second network structure with covalent links not shown here for didactic reasons.

It is not surprising that diabetes mellitus has been one of the first and most intensively explored pathology for such carrier systems, since the disease is of high socioeconomic relevance, and glucose levels as a predictive physiological marker are easily detectable in the blood circulation. Glucose-responsive systems can be categorized by their sensing principles: (i) the enzymatic oxidation of glucose by glucose oxidase (GOx) to glucuronic acid (the pH drop affects pH-sensitive polymers), (ii) the binding of glucose with the lectin concanavalin A (ConA) (competitive binding removes the physical crosslinks of hydrogels), and (iii) the reversible covalent bond formation between glucose and boronic acids (Figure 17) [233,234]. Various types of typically pH-sensitive chain segments have been used in hydrogels in combination with GOx, including copolymers of HEMA-DMAEMA (dimethylaminoethyl methacrylate) [235,236], HPMA-DEAEMA (diethylaminoethyl methacrylate) [237], and poly(methacrylic acid-*g*-ethylene glycol) [238]; additionally, approaches based on heterogeneous membranes with switchable pores based, e.g., on PAAc [239] or PMAAc-PNIPAAm [240] are reported. Additionally, for ConA, several natural and artificial sugar-like structures were explored to build hydrogels, and their interactions with the four binding sites of ConA were employed, e.g., glycogen [241], poly(2-glucosyloxyethyl methacrylate) (PGEMA) [242], mPEG-*g*-poly(vinylpyrrolidone-*co*-acrylic acid) (mPEG-PVPAAc) [243], poly(acrylamide-*co*-allylglucose) [244], or PNIPAAm [245,246]. In addition to bulk hydrogels, crosslinking via ConA has also been used inside microgel particles formed via emulsion templating [247]. As the third concept, the reversible binding of polyols to boronic acid has attracted more continuous attention. In some pioneering work, pendent phenylboronic acid moieties were copolymerized, e.g., with *N*-vinyl-2-pyrrolidone (NVP), to build hydrogels via complexation with polyols (e.g., PVA) that were destructed with excess glucose [248,249]. Alternatively, copolymers with *N*-(2-dimethylaminopropyl)acrylamide) (DMAPAA) can shift their LCST in the presence of glucose [250]. Despite the low specificity of phenylboronic acid for glucose, and thus, possible interference by other types of molecules, this concept was also applied to gel particles [251,252], micellar systems [253], and mesoporous silica particles with pore-blocking shells [254]. While there is a variety of material concepts for glucose-triggered insulin release, chances for pharmaceutical commercialization may also depend on safety aspects, including the potential immunogenicity of plant-derived proteins such as ConA, or on the benefits of such carriers compared to existing insulin dosage schemes in a saturated market, which is moving towards needle/injection-free applications. Still, there may be applications for glucose-sensitive gels beyond insulin release, e.g., in the case of a diabetic wound therapy, where proangiogenic desferrioxamine was released from a phenylboronic acid-containing gel via the collaborative/consecutive effects of several factors associated with conditions of the wound bed (hyperglycemia, reactive oxygen species, and matrix metalloproteinases) [255]. Phenylboronic acid-derived crosslinks formed, e.g., with catechols, can sense other stimuli in addition to glucose, such as pH or *p*-dihydroxyphenolic substances such as dopamine [256,257]; this broadens the possibility of applicable stimuli, while at the same time, illustrating potential sources of interference in an in vivo setting.

**Figure 18:**
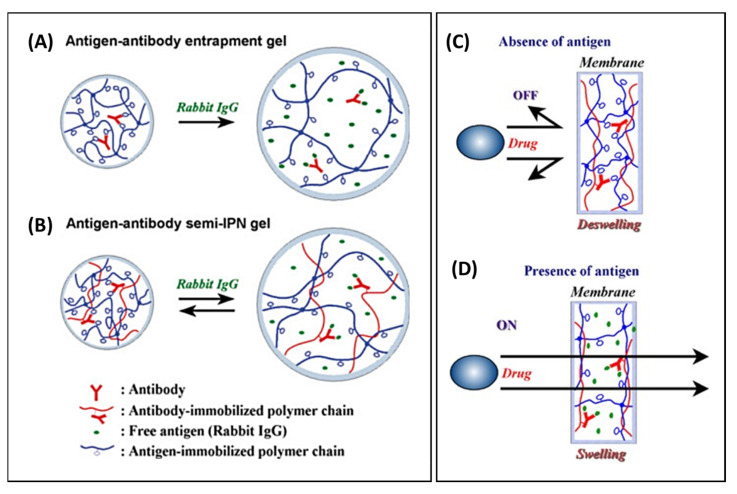
Schematic concepts of responsive release systems with switches based on antigen–antibody affinity. (**A**,**B**) Hydrogels network architectures resulting in (**A**) non-reversible and (**B**) reversible responses (gel swelling) upon exposure to the antigen (here rabbit IgG was used as antigen). Reprinted with permission from [265]. Copyright 2009, Wiley Periodicals, Inc. (**C**,**D**) Permeation pattern of a drug through an antigen–antibody membrane in response to the (**C**) absence and (**D**) presence of a target antigen. Reprinted with permission from Springer Nature Customer Service Center GmbH: Springer Nature [264], Copyright © 2022, The Society of Polymer Science, Japan.

Due to their high specificity, antigen–antibody binding may satisfy the demand for highly specific switches in materials for biosensing and release technologies [258]. In principle, aptamers may also be used for switches via affinity binding [259]. Based on the specificity of these interactions, complex biological markers released in vivo may be sensed, ranging from inflammatory factors to, e.g., hormones. Furthermore, antibodies directed against threats attacking the body from outside may be part of future release systems capable of providing, e.g., antidotes at a time when a certain complex biohazard is sensed. Thus far, either full antibodies [260] or their antigen binding fragments [261] have been used as switches, e.g., via physical entrapment or chemical conjugation of the antibody and/or the antigen to the network (Figure 18). Reversibility of the swelling may be achieved when both species, antigen and antibody, are fixed in the hydrogel such as in semi-interpenetrating networks [260]. While many studies involved large-molecule model antigens [262], comparatively small chemical compounds can also serve as antigens [263] and may be used to at least alter the permeation profiles of macromolecules through hydrogel matrices [264]. As holds true for all biopharmaceuticals, stability and costs may decide whether such systems could expand beyond the experimental state.

For polymer responses to the in vivo signaling of “danger”, reactive oxygen species (ROSs) have been recognized as a physiological factor of intracellular communication, as well as a relevant danger signal expressed at high levels by cells under stress conditions, triggering, e.g., immunological responses. A substantial number of ROS-sensitive moieties are known, with some of them being relevant in a biological setting [266]. Suggested concepts for use in polymers and delivery systems include, for instance, the decomposition of peroxalate ester linkages in copolyoxalate nanoparticles releasing vanillyl alcohol—its anti-inflammatory comonomer [267]. Via the scission of phenylboronic acid esters as side chains of PNIPAAm copolymers, an LCST shift was mediated for the opening of clogged pores of mesoporous silica nanoparticles [268]. The backbone cleavage of thioketal linkages of poly(1,4 phenyleneacetone dimethylene thioketal) nanoparticles [269], the oxidation of thioethers to more hydrophilic sulfoxides/sulfones for the enhanced swelling of hydrophobic polymer network nanoparticles [270], and the disassembly of micelles from initially amphiphilic block copolymers [271] are some further examples of employed principles based on redox-sensitive moieties. Additionally, the splitting of ester bridges was used in theranostic agents built by oxalic acid to release both the drug and a diagnostic dye [271]. Disulfide bonds are another example of linkages, and have been extensively explored in various facets to facilitate polymer degradation in redox conditions [272,273]. For instance, micelles with cores crosslinked by disulfide bonds released cytotoxic drugs in vitro in the presence of glutathione (S-S bond cleavage) depending on the core architectures built in the crosslinking step, which can be affected by the nature of the hydrophobic substituents [274]. Positive feedback, i.e., the amplification of reaction speed by increasing the access of ROSs to the material, can be a characteristic feature in ROS-sensitive polymers with hydrophilicity shifts.

### 3.4. Responsiveness to Specific Cells

Different cell types are characterized and distinguishable by their function and morphology, which are a consequence of the expression of proteins and other molecules within and at the surface of the cells. One path to create responsiveness of materials to cells can work through solute molecules present within the cells or being expelled by the cells, as discussed in previous sections of this review. As examples, intracellular pH changes within endosomal compartments or the release of ROSs may be recalled. Cytokines are another larger group of secreted molecules, which are part of a more specific type of intercellular communication, particularly in immune cells. Based on the concepts highlighted above, cytokines can be considered as potential stimuli for hydrogel networks, in which they might compete with dimeric derivatives acting as junctions, and thereby allow for increased swelling/drug release.

Characteristic markers that help distinguishing cell types are molecules expressed at the cell surfaces, which typically possess distinct functions in cellular communication. Those surface-expressed molecules can be a second path to realizing cell-responsive materials. Clusters of differentiation on immune cells are examples of potential cell markers that can be recognized by antibodies/antibody fragments/aptamers, etc. Other examples are targeting units based on glycoproteins with terminal galactose or motives with triantennary N-acetylgalactosamine residues, which can bind to asialoglycoprotein receptors highly expressed on hepatocytes. Along with the discussion of Figure 7, the binding of HA to CD44 has already been mentioned as a delivery strategy. When combining stimuli-sensitive polymers and cell-targeting motives in a carrier system, the approximation of a stimuli-sensitive carrier to the cell through the targeting motive, and thus, the responsiveness of the carrier’s polymer matrix to cell-secreted stimuli or stimuli co-expressed at the cell surface may be enhanced. The use of polymer conjugates has also been proposed for diagnostic and bioseparation purposes, e.g., through in situ coupling of a temperature-sensitive polymer tail to antigen–antibody complexes that allowed theprecipitation, and thus, enrichment the antigen of interest [275].

Enzymes are among the most prominent groups of triggers that can, typically irreversibly, change polymer structures, and thereby trigger a predefined response (see Section 3.2). For instance, programmed SMP networks containing digestible chain segments for shape fixation (e.g., polyesters sensitive to lipase), in addition to biostable segments defining the permanent shape, can perform shape-recovery in response to cellular contact [276]. In addition, secreted matrix metalloproteinases, cathepsin B as a lysosomal protease, caspases as cytosolic proteases, and several other enzymes have been considered for enzyme-responsive polymeric materials, with, in some cases, reasonable specificity to distinct peptide motives used as cleavable peptide linkers in polymeric materials [277].

## 4. Future Considerations and Conclusions

It is doubtless that switchable materials are one of the main driving forces for new functions being implemented in experimental polymer-based drug-delivery systems. Substantial opportunities may arise when such systems allow outside-control or even self-control of drug dosing according to the present spatial or temporal demand for each individual patient. It can be expected and should be encouraged that research will concentrate even more on in vivo-derived and, importantly, in vivo-relevant stimuli. At the same time, this leads to challenges associated not only with the complexity of multi-component carrier systems in terms of regulatory issues, but also with the hurdles of academic research in the progression of promising concepts towards clinical studies for overcoming common skepticism of their clinical relevance. In this context, it is highly relevant for the community to critically evaluate the applicability of material concepts arising from polymer science in pharmaceutical sciences, in the context of conditions that are present or acceptable in a physiological or pathophysiological setting.

## Data Availability

The data are available within the article.
